# McComedy: A user-friendly tool for next-generation individual-based modeling of microbial consumer-resource systems

**DOI:** 10.1371/journal.pcbi.1009777

**Published:** 2022-01-24

**Authors:** André Bogdanowski, Thomas Banitz, Linea Katharina Muhsal, Christian Kost, Karin Frank

**Affiliations:** 1 Osnabrück University, Department of Ecology, School of Biology/Chemistry, Osnabrück, Germany; 2 Helmholtz-Centre for Environmental Research – UFZ, Department of Ecological Modelling, Leipzig, Germany; 3 Osnabrück University, Institute for Environmental Systems Research, Osnabrück, Germany; 4 iDiv – German Centre for Integrative Biodiversity Research, Halle-Jena-Leipzig, Germany; University of Minnesota, UNITED STATES

## Abstract

Individual-based modeling is widely applied to investigate the ecological mechanisms driving microbial community dynamics. In such models, the population or community dynamics emerge from the behavior and interplay of individual entities, which are simulated according to a predefined set of rules. If the rules that govern the behavior of individuals are based on generic and mechanistically sound principles, the models are referred to as next-generation individual-based models. These models perform particularly well in recapitulating actual ecological dynamics. However, implementation of such models is time-consuming and requires proficiency in programming or in using specific software, which likely hinders a broader application of this powerful method. Here we present *McComedy*, a modeling tool designed to facilitate the development of next-generation individual-based models of microbial consumer-resource systems. This tool allows flexibly combining pre-implemented building blocks that represent physical and biological processes. The ability of *McComedy* to capture the essential dynamics of microbial consumer-resource systems is demonstrated by reproducing and furthermore adding to the results of two distinct studies from the literature. With this article, we provide a versatile tool for developing next-generation individual-based models that can foster understanding of microbial ecology in both research and education.

## Author summary

Microorganisms such as bacteria and fungi can be found in virtually any natural environment. To better understand the ecology of these microorganisms–which is important for several research fields including medicine, biotechnology, and conservation biology–researchers often use computer models to simulate and predict the behavior of microbial communities. Commonly, a particular technique called individual-based modeling is used to generate structurally realistic models of these communities by explicitly simulating each individual microorganism. Here we developed a tool called *McComedy* that helps researchers applying individual-based modeling efficiently without having to program low-level processes, thus allowing them to focus on their actual research questions. To test whether *McComedy* is not only convenient to use but also generates meaningful models, we used it to reproduce previously reported findings of two other research groups. Given that our results could well recapitulate and furthermore extend the original findings, we are confident that *McComedy* is a powerful and versatile tool that can help to address fundamental questions in microbial ecology.

## Introduction

Microbial communities are pervasive across all ecosystems and most often essential for their functioning [1,2]. However, the vast taxonomic diversity of their members, manifold interactions within communities and between microorganisms and their environments, as well as heterogeneities (e.g. in composition and functioning) across spatial and temporal scales pose a major challenge to understand their ecology [1,2,3–5]. On the other hand, a better sense of how microbial communities assemble and respond to environmental conditions is essential to fuel advance in various research fields such as medicine, biotechnology, and climate change research [6–8].

Microbial community dynamics usually involve metabolic interactions such as the exchange of and competition for resources [9,10]. Focusing on those interactions, microbial communities together with the resources can be viewed as consumer-resource systems. Traditionally, consumer-resource systems are modeled using differential equations for the densities of consumer and resource species at the level of populations [11,12]. Such population-level equations are still applied in microbial ecology [13–15], but recent research of microbial consumer-resource systems is increasingly concerned with the dynamics within populations, particularly when a spatial component needs to be explicitly considered [16–19]. Such spatially explicit approaches can provide insight on how localized processes (e.g. cross-feeding in a structured environment [16,18]) shape the community on a larger scale. For that, individual-based models (IBMs) are widely applied [20].

IBMs are commonly used to investigate the dynamics of populations or communities by simulating individual entities, which in ecology usually represent individual organisms [21,22]. The dynamics of populations and communities then emerge from the simulated behavior of these individuals. This bottom-up approach has been shown to be particularly useful for modeling complex systems, where individuals exhibit trait variation, adaptive behavior, or localized interactions [23,24].

The relatively well-understood nature of individual microorganisms in terms of movement, metabolism, and reproduction (as opposed to the more complex dynamics at the level of populations and communities) makes microbial systems particularly well-suited for simulation in IBMs. For this reason, IBMs are frequently applied to analyze different aspects of microbial ecology and evolution [20]. The simulation model results can be analyzed on different levels of organization, ranging from below (e.g. metabolic networks within individual microorganisms [19,25,26]), at (e.g. movement trajectories of individuals) and above the level of individuals (e.g. spatial distributions of entire populations or community compositions [16–18]). In addition, the resulting data can be directly compared to results derived from experiments, thus making IBMs very powerful to link empirical observations with theory.

IBMs can be distinguished between traditional ones and so-called next-generation IBMs [27]. Traditional IBMs are designed and parametrized on the basis of site-specific measurements (e.g. the interaction of two species is modeled according to their co-occurrence in the modeled ecosystem). This makes these models non-generic and non-transferable to other environments [27]. Next-generation IBMs overcome this drawback by constructing the individuals’ behavior from generic submodels that are based on well-understood principles from physics, chemistry, physiology, and evolutionary biology [24,27]. This mechanistic approach increases the propensity of the models to capture the organization and functioning of the real system (i.e. *structural realism*) rather than only matching empirically observed patterns [24]. In microbial ecology, this is reflected in several IBMs (e.g. [19,28–30]), which result in strikingly realistic model behavior and a thorough understanding of ecological mechanisms. Besides providing specific insights in their respective fields of application, these models demonstrate the general potential of next-generation IBMs for microbial ecology. However, building and using next-generation IBMs usually requires good knowledge in programming or proficiency with specific software tools, which hinders a more widespread application by microbial ecologists. An easy-to-use framework that facilitates the development of such IBMs from pre-implemented, tested, generic and mechanistically sound submodels could therefore contribute significantly to the field.

Here we present the modeling tool *McComedy* (**M**i**c**robial **Co**mmunities, **Me**tabolism, and **Dy**namics), which constitutes a framework for individual-based modeling of microbial consumer-resource systems. A central idea of this framework is to provide generic submodels based on biological and physical principles, which we refer to as *process modules* and which can be combined and parametrized in a user-friendly graphical interface, resulting in ready-to-use next-generation IBMs. We tested the validity of our approach by using *McComedy* to implement two specific IBMs corresponding to two different studies of spatial and evolutionary dynamics of microbial communities, which involved both experiments and IBMs. For both cases, we demonstrate that the respective model constructed with *McComedy* was able to robustly reproduce the general results and capture the essential mechanisms underlying the microbial community dynamics in the original studies. Furthermore, we demonstrate how *McComedy* can be used for tackling open research questions by extending the two original studies with additional insights.

## Results

### McComedy

**Fig 1:**
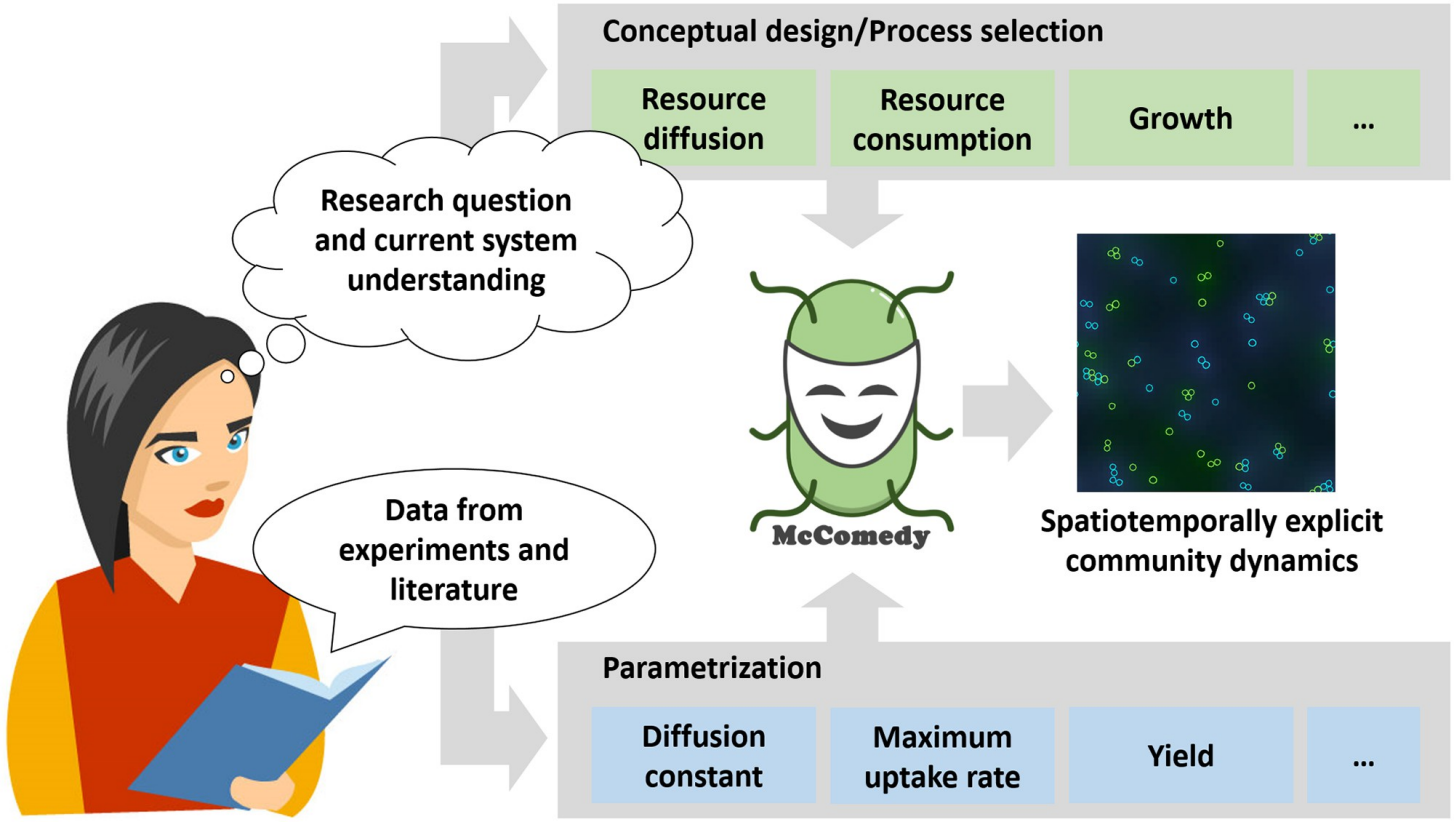
Intended workflow when using *McComedy*. The modeler designs an individual-based model (IBM) by selecting process modules under consideration of the research question and the current understanding of the system. The parameter values that are necessary for the simulation of the selected processes are set by the modeler, e.g. according to experimental data or literature. The resulting IBM generates spatiotemporally explicit data of the modeled microbial system.

*McComedy* is an open-source modeling tool for developing and using IBMs of microbial communities, with a focus on consumer-resource interactions and their implications for the functioning of the corresponding communities. This tool was developed to facilitate fast and user-friendly operation as well as to grant high flexibility in model design (Fig 1). The software can be downloaded from https://git.ufz.de/bogdanow/mccomedy, where also the source code and a tutorial on how to get started are provided. To create a new IBM, the user can select several process modules, which implement biological and physical processes of relevance for microbial consumer-resource systems such as consumption or production of resources, resource diffusion through the environment, and growth of individual microorganisms. Next, parameter values of the selected process modules can be defined according to the specifics of the modeled system on the basis of empirical observation or literature. Subsequently, simulations are executed and spatially explicit data on the modeled system at discrete time points is generated.

A specific IBM created with *McComedy* describes a three-dimensional environment in which individual microorganisms (in *McComedy* referred to as *microbes*) and resources interact. Microbes are modeled as individual objects with spherical shapes and continuous positions in the environment. Specific types can be defined that differ in certain traits, such as their metabolic requirement or growth parameters. Resources are not modeled as individual particles but instead as concentrations in each grid cell of a three-dimensional grid covering the simulated environment. Resources are different metabolites that can be consumed or produced by the microbes. If necessary, the three-dimensional environment can be reduced to represent two dimensions by constraining the third dimension to just one layer of grid cells.

Over the simulated time span, microbes and resources are subject to the modeled processes. These processes are encapsulated in so-called process modules, which mediate direct and indirect interactions among microbes and between microbes and the resources. Each process module simulates one component of the system dynamics, such as microbial growth or resource diffusion. In order to facilitate a flexible yet functional model design, each process module is implemented based on generic principles, which means that no *ad-hoc* assumptions are made for particular model applications. Instead, the dynamics of each modeled microbial system emerge entirely from the same pool of generic principles. For example, the process module *Growth* transforms consumed resources into biomass under consideration of a yield to be defined (cf. McComedy ODD protocol (S1 Text), 7.2.6 Growth). The module *Replication* divides a microbe individual into two once a critical biomass has been reached (cf. McComedy ODD protocol (S1 Text), 7.2.11 Replication). These processes are mechanistically valid regardless of the specific modeled system and are therefore preferable to alternatives, such as imposed rules or *ad-hoc* assumptions (e.g. a microbe replicating by chance when it is close to resources). We use the term *generic principles* (instead of *first principles*, which is also common in the literature [24]), because we do not claim that our processes are completely described by scientific laws, as we also use reasonable simplifications if we consider them mechanistically sound. *McComedy* does not allow for imposing higher-level processes (e.g. spatial pattern formation or density-dependent regulation of population size) as such dynamics are supposed to emerge from the generic process modules.

The graphical user interface of *McComedy* supports a fast and user-friendly model development. The user is guided through different development stages, starting with the selection of process modules. According to the selection, *McComedy* shows tables containing the required parameters with editable default values. The user can also specify lists of values for single parameters and *McComedy* will run simulations for every combination of these parameter values. Moreover, the user can control technical settings such as the number of replicates and the configuration of the model output.

The model output is generated separately for each individual simulation, in order to facilitate comparative analyses with regard to parameter variations as well as variance analyses due to stochasticity. For each simulation, the state of each microbe and resource grid cell is written into result files at predefined time intervals. The aforementioned state includes spatial coordinates, biomass, microbial type, resource concentration, as well as other properties, which allow not only for a highly-resolved and spatially explicit model analysis, but also for a direct statistical comparison with a variety of empirical data (e.g. growth kinetics, spatial patterns, functional responses, etc.).

The computation time for a simulation depends mostly on the size of the simulated environment, the number of microbes included, the time step lengths of the process modules, the termination condition, and the hardware used. Simulating a microbial community for 10 virtual hours on a regular computer can take between few minutes and several days. We provide an estimate of reference computation times on a current standard computer for different representative parameter choices in S2 File.

For further details on the implementation and use of *McComedy* please consult the Methods section as well as the ODD protocol (S1 Text).

To demonstrate that the IBMs built with *McComedy* can capture and serve to analyze the dynamics of specific microbial systems, *McComedy* was used to reproduce the outcomes of two exemplary studies of microbial systems. The studies were chosen from the literature based on the close correspondence of their research questions to *McComedy*’s intended field of application. Thus, both studies assess spatial structuring in microbial communities as a consequence of consumer-resource interactions. We compared the outcomes of *McComedy* with the empirical and modeling results of the original studies. In the following, we show how *McComedy* can help to analyze and compare the respective results and how it can provide additional insight into the underlying mechanisms.

### Example 1: Spatial organization model (Mitri *et al*. [17])

To fully understand the functioning of microbial communities, it is essential to identify the drivers of spatial structuring and the diversity in their assemblage. In this context, Mitri *et al*. [17] conducted experiments with bacteria to assess whether resource limitation leads to spatial separation of different strains in an initially well-mixed, growing colony. The authors used an IBM to identify the ecological mechanisms underlying their experimentally observed results.

**Fig 2:**
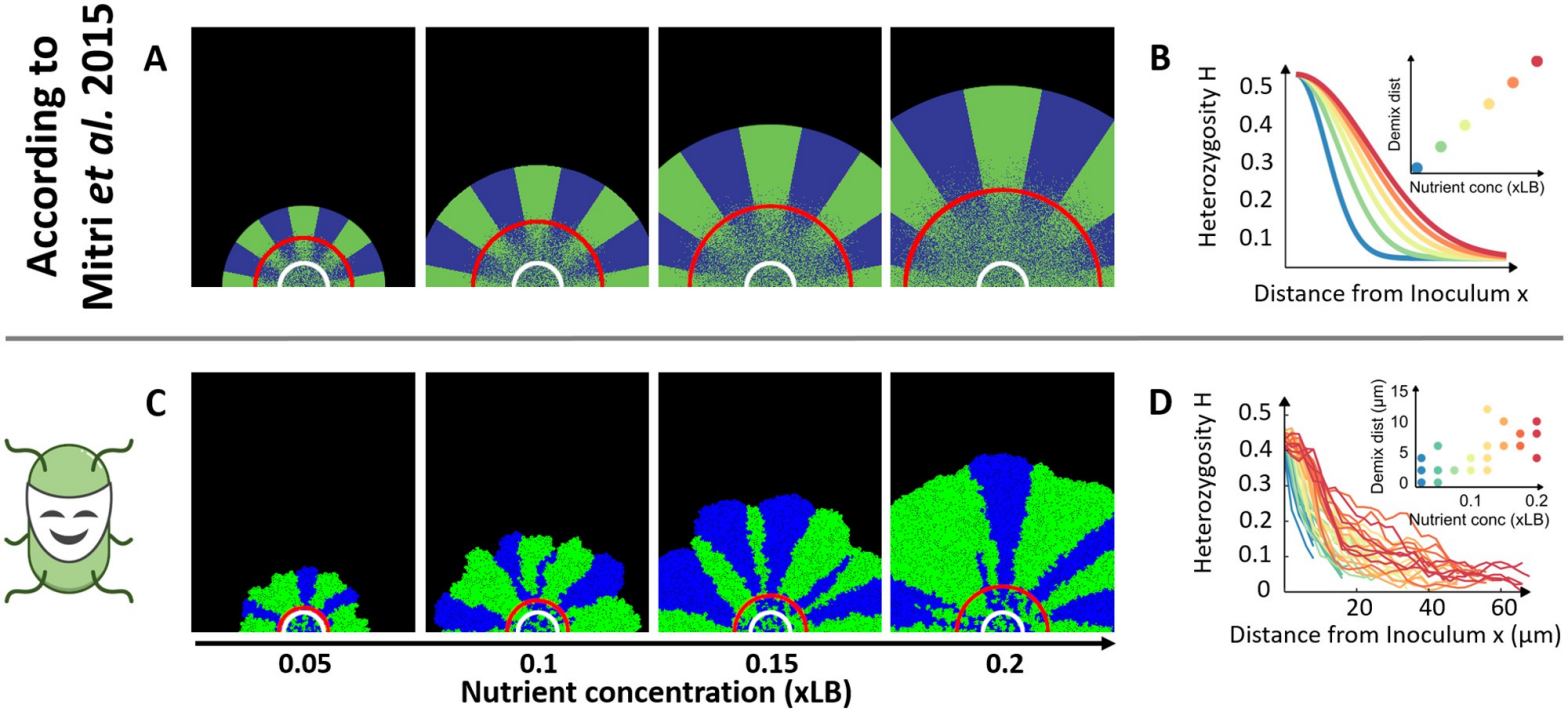
*McComedy* can reproduce the results of experiments and simulations by Mitri *et al*. [17] both quantitatively and qualitatively. Top views on colonies at different initial resource (nutrient) concentrations and degree of heterozygosity over the distance to the inoculum. The unit xLB is defined as the fold-concentration of LB medium. Blue and green colors on colony images indicate the two bacterial strains. White circles on the colony images indicate the inoculum. Red circles indicate where the demixing area begins. Analyses with *McComedy* were conducted after 45 simulated hours of growth. **A**: Stylized recreation of top views on colonies at different resource concentrations according to Figures 2a and 4a in [17]. **B**: Stylized recreation of the heterozygosity over distance from inoculum and corresponding demixing distances at different resource concentrations according to Figures 2c and 4b in [17]. Axis labels of distances are not shown as they varied between experimental and simulation results and were of no consequence for the qualitative pattern. **C**: Representive top views on colonies at different resource concentrations in the *McComedy* IBM. **D**: Heterozygisity over distance from inoculum and estimated demixing distances at different resource concentrations in the *McComedy* IBM. Images **A** and **B** were recreated due to copyright issues. Refer to Figures 2 and 4 in [17] to view the original data.

In the experiment, two droplets of two differently labeled strains of *Pseudomonas aeruginosa*, visually discriminable by green and blue color, were spotted on nutrient-poor agar. After two weeks of incubation, the colony had grown in size and exhibited a strong pattern of intermixing among the two strains in the center, yet a clear separation of green and blue bacteria in the outer rim of the colony. Increasing the initial resource concentration in the agar led to an increased demixing distance, defined as the distance between the initial inoculum and the region of spatial separation (Fig 2A, Figure 2a in [17]). Quantitatively, the observed spatial structure of the colony was assessed by measuring the degree of heterozygosity (i.e. how much the two strains were intermixed in a given location) across the colony (Fig 2B, Figure 2c in [17]). The demixing distance corresponds to the distance from the initial inoculum, at which heterozygosity showed the steepest decrease.

The correlation of resource concentration and demixing distance was hypothesized to be attributable to the varying resource accessibility at the periphery of the colony. At high resource concentrations, more resources diffuse into the colony, which support the growth of a higher number of bacterial cells, thus reducing the chance of excluding one strain from growth at a given location. Analogously, at low resource concentrations, growth of fewer bacterial cells is supported at the edge of the colony, resulting in a more immediate loss of the local diversity. In population genetics, this mechanism is known as the bottleneck effect [31]. Mitri *et al*. [17] applied an IBM to recapitulate the empirical pattern (Figure 4a and 4b in [17]). In agreement with the hypothesis, also in the model the demixing distance increased with increasing initial resource concentrations. Based on the analysis of their model, the authors thus concluded that the bottleneck effect in an expanding colony is indeed the mechanism that most likely explains spatial separation of bacteria under resource-limited conditions.

To validate *McComedy*, we created an IBM to recapitulate the results presented by Mitri *et al*. [17]. In accordance with the original system, the model was specified with two types of bacteria having exactly the same properties (except for their color) and a resource at varied, initially homogeneous concentrations in a two-dimensional environment. Process modules were selected to account for resource diffusion, resource consumption, microbial growth, and replication. A detailed description of the model implementation is provided in the Methods section and a complete list of the selected process modules and parameter values is available in S1 File.

The model simulations generated very similar bacterial community dynamics compared to the original study (Fig 2C and 2D) and also the spatial organization of the two strains (Fig 2C) qualitatively matched those described by Mitri *et al*. [17] (Fig 2A and Figures 2a and 4a in [17]). The resulting colonies showed a clear separation (demixing) of the two strains towards the edge of the colony, whereby the demixing distance also increased with higher resource concentrations. For a quantitative analysis of the demixing dynamics in response to different initial resource levels, the measure of heterozygosity was calculated based on the exact position of each single bacterial individual in the simulations (Fig 2D) as was done for the original model results (Figure 4b in [17]). This analysis showed that heterozygosity dropped from approximately 0.5 (highly mixed strains) at the inoculum to almost zero (segregated strains) at the edge of the colony. Moreover, the demixing distance increased with initial resource concentrations. Quantitatively, both simulation models do not precisely match the experimental data and show slight discrepancies between each other, which may originate from different implementation details or choices of parameter values. However, the consistent qualitative response of the spatial pattern to the varied resource concentrations demonstrates that also the new model is well-suited to study the mechanisms generating such patterns. This is facilitated by *McComedy’s* capabilities to observe and quantify the characteristics of spatiotemporal colony dynamics that emerge from suites of different scenarios.

**Fig 3:**
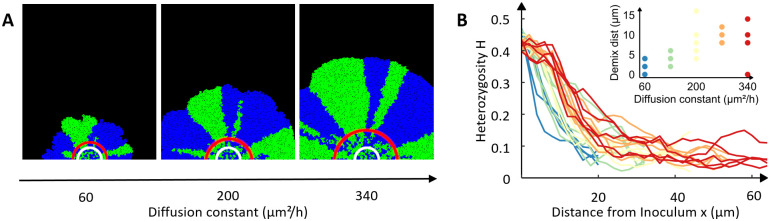
Increased diffusion resulted in an increased demixing distance. Simulations were performed with *McComedy*. Analysis after 39 simulated hours of growth. **A**: Top views on representative colonies as simulated using *McComedy* using different resource diffusion constants. Blue and green colors on colony images indicate the two bacterial strains. White circles on the colony images indicate the size of the inoculum. Red circles indicate where the demixing area begins. **B**: Heterozygosity over distance from inoculum and estimated demixing distance at different resource diffusion constants.

To further test the potential of *McComedy* for understanding mechanisms operating in microbial consumer-resource systems, we used it to simulate colony growth under different resource diffusion constants, while keeping the initial resource concentration constant. This type of analysis should additionally corroborate the explanation by Mitri *et al*. [17], which attributes the spatial separation to the bottleneck effect. Here we hypothesized that increasing the resource diffusion constant should have an effect that is similar to increasing the initial resource concentration. Indeed, simulating increased rates of diffusion revealed that resources diffused deeper into the colony, thus resulting in less spatial segregation of both strains and an increased demixing distance (Fig 3). These results confirm that the bottleneck-effect drives the separation of the two populations and that *McComedy* is a powerful tool to investigate the spatiotemporal dynamics and mechanisms underlying experimental observations.

### Example 2: Cooperation model (Momeni *et al*. [16])

The second example concerns research on the maintenance of cooperation in spatially structured environments, where pairs of individual microorganisms can interact repeatedly (as opposed to a well-mixed, spatially unstructured environment). In this context, it is important to understand how metabolic interactions affect the spatial organization of resident strains and thus the distribution of different strategists within microbial communities. Momeni *et al*. [16] performed experiments with yeast strains to investigate how spatial self-organization affects the abundance of cooperative and non-cooperative strains. For this, they used synthetically engineered cooperative and non-cooperative strains of *Saccharomyces cerevisiae*, of which the former two strains provided the resources lysine and adenine to the community. In their study, the observation that cooperators intermix, while non-cooperators spatially segregate, was explained using an IBM.

**Fig 4:**
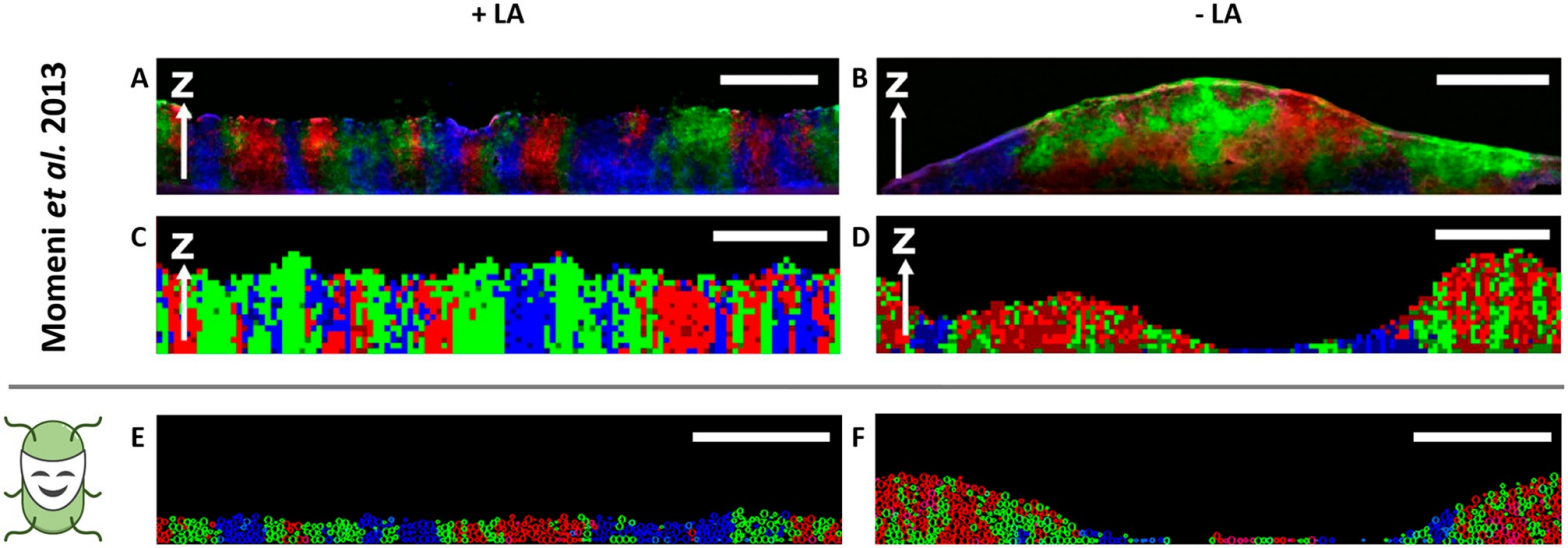
*McComedy* reproduces qualitative results of experiments and simulations by Momeni *et al*. [16]. Vertical cross-section views on layers of yeast cells grown on media supplemented with lysine and adenine (+ LA) and on media without these resources (- LA). Red and green color indicates the two cooperative yeast strains, blue color indicates the non-cooperative yeast strain. Simulations performed with *McComedy* were visualized after 6 generations. **A, C, E**: Representative cross-sections of yeast cells grown with supplemented lysine and adenine (+LA) in the experiment, original IBM, and *McComedy* IBM, respectively. **B, D, F**: Representative cross-sections of yeast cells grown without lysine and adenine (-LA) in the experiment, original IBM, and *McComedy* IBM, respectively. Scale bar: 100 μm. Images **A, B, C, D** adapted from [16].

In particular, the authors designed a system with three strains of *Saccharomyces cerevisiae*, two complementary cooperators and one non-cooperator. One cooperator strain $G_{\to L}^{\leftarrow A}$ required adenine and released lysine upon cell death, while the other cooperator $R_{\to A}^{\leftarrow L}$ required lysine and continuously released adenine. In contrast, the non-cooperating strain *C*^←*L*^ also required lysine for growth, yet did not release any resource to enhance the growth of other cells. This latter strain gained a fitness advantage from not sharing resources. After mixing the three strains and plating them on agar at low density, individual yeast cells formed colonies that increased in diameter, until the entire agar plate was covered after which the yeast cells started to grow upwards. During this process, the two cooperating strains intermixed with each other, grew well, and formed a thick layer, whereas the non-cooperators spatially segregated from the cooperators and only formed a thin layer of cells (Fig 4B). In a control experiment, the growth medium was supplemented with adenine and lysine, such that the two cooperators could grow independently of the cooperation of their corresponding partners. Under these conditions, cooperation turned into competition for space and other limiting resources. As a consequence, none of the strains intermixed to a significant extent and the thickness of the microbial layer was almost uniform, independent of which strain formed it (Fig 4A).

**Fig 5:**
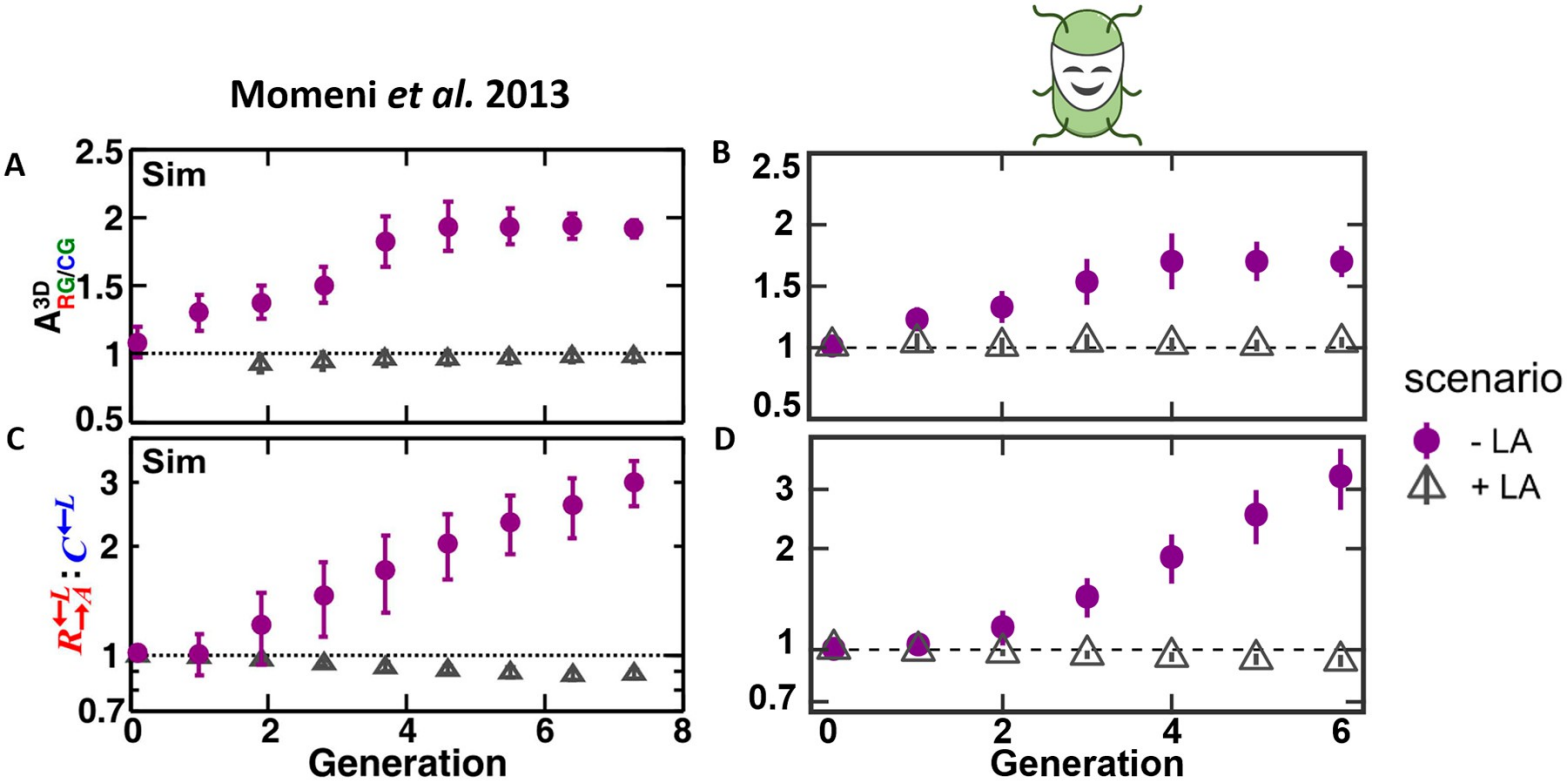
*McComedy* reproduces quantitative results of simulations by Momeni *et al*. [16]. The quantitative metrics were assessed for yeast cells grown on media supplemented with lysine and adenine (+ LA) and on media without these resources (- LA). **A, B**: Association index of the two cooperative strains ($R_{\to A}^{\leftarrow L}$ with $G_{\to L}^{\leftarrow A}$) and the non-cooperators **C**^**←L**^ in the original IBM and *McComedy* IBM, respectively. **C, D**: Abundance ratio between the cooperators $R_{\to A}^{\leftarrow L}$ and the non-cooperators **C**^**←L**^ in the original IBM and *McComedy*, respectively. Note the logarithmic scales of the vertical axes. Images **A, C** adapted from [16].

An IBM was used to recapitulate these experimental results and examine the mechanism that explains the observed exclusion of non-cooperating types. The simulations robustly reproduced the partner intermixing of the two cooperative strains (Fig 4C and 4D). Moreover, it was shown that the spatial association of the cooperators $G_{\to L}^{\leftarrow A}$ with their partners $R_{\to A}^{\leftarrow L}$ increased over time compared to their association with the non-cooperating strain *C*^←*L*^. These association differences were quantified by computing the association index $A_{RG/CG}^{3D}$, which is the ratio between the frequencies of individuals of type $G_{\to L}^{\leftarrow A}$ in the direct vicinity of individuals of type $R_{\to A}^{\leftarrow L}$, and in the direct vicinity of individuals of type *C*^←*L*^ (Fig 5A). Furthermore, the abundance of the cooperator $R_{\to A}^{\leftarrow L}$ relative to the corresponding non-cooperator *C*^←*L*^ (both of which compete for lysine) increased (Fig 5C). The following mechanism drove the observed spatial self-organization: distinct populations that reciprocally provide each other with localized benefits are expected to intermix as individual yeast cells grow best in the vicinity of a cooperating partner [32]. By the same logic, populations that provide no localized benefits to the community are expected to segregate. The IBM demonstrated that this mechanism alone was sufficient to generate the observed spatial patterns and no additional rules implemented by the modeler such as e.g. partner recognition or positive chemotaxis were required.

To verify whether *McComedy* can reproduce the results of Momeni *et al*. [16], a corresponding model of a microbial system with two cooperating and one non-cooperating yeast strains and two resource types was implemented in *McComedy*. Simulations were performed in a three-dimensional environment and yeast cells were initially distributed on a plane at the bottom. Process modules were selected to account for the production, release, diffusion, and consumption of resources, microbial growth, replication and mortality, and a weak gravitational force that kept the yeast cells at the bottom of the environment. Other resources than lysine and adenine were not explicitly modeled but assumed to be not limiting and constantly available for microbial uptake. This allows for the production of lysine or adenine, respectively, also for non-growing individuals. A detailed description of the model implementation is provided in the Methods section and a complete list of the selected process modules and parameter values is available in S1 File.

The IBM created with *McComedy* succeeded in qualitatively reproducing the self-organized pattern observed in both the original IBM and the experimental setup. In the competitive scenario with additional adenine and lysine provided (+ LA), the microbial layer consisted of strongly separated yeast strains, which exhibited uniform thickness (Fig 4E). In the scenario without additional resource providing (- LA), cooperating partners intermixed and formed thick layers, whereas non-cooperators were spatially excluded from the cooperative benefit and only formed thin layers (Fig 4F). For a quantitative comparison, the two measures from the original study (i) association index and (ii) ratio of the abundances of $R_{\to A}^{\leftarrow L}$ and *C*^←*L*^ were calculated based on the new simulation results.

Both measures match very well between the two models. The association index increases in both cases initially, before plateauing after approximately four generations between values of 1.5 and 2 (Fig 5A and 5B). The ratio of abundances of $R_{\to A}^{\leftarrow L}$ and *C*^←*L*^ increases in both models exponentially (Fig 5C and 5D, note the logarithmic vertical axes). Both the original model and the new *McComedy* model fit the empirical evidence, as the experimental setup was evaluated once after six to eight generations, showing an increased intermixing of cooperators (cf. original study [16]).

**Fig 6:**
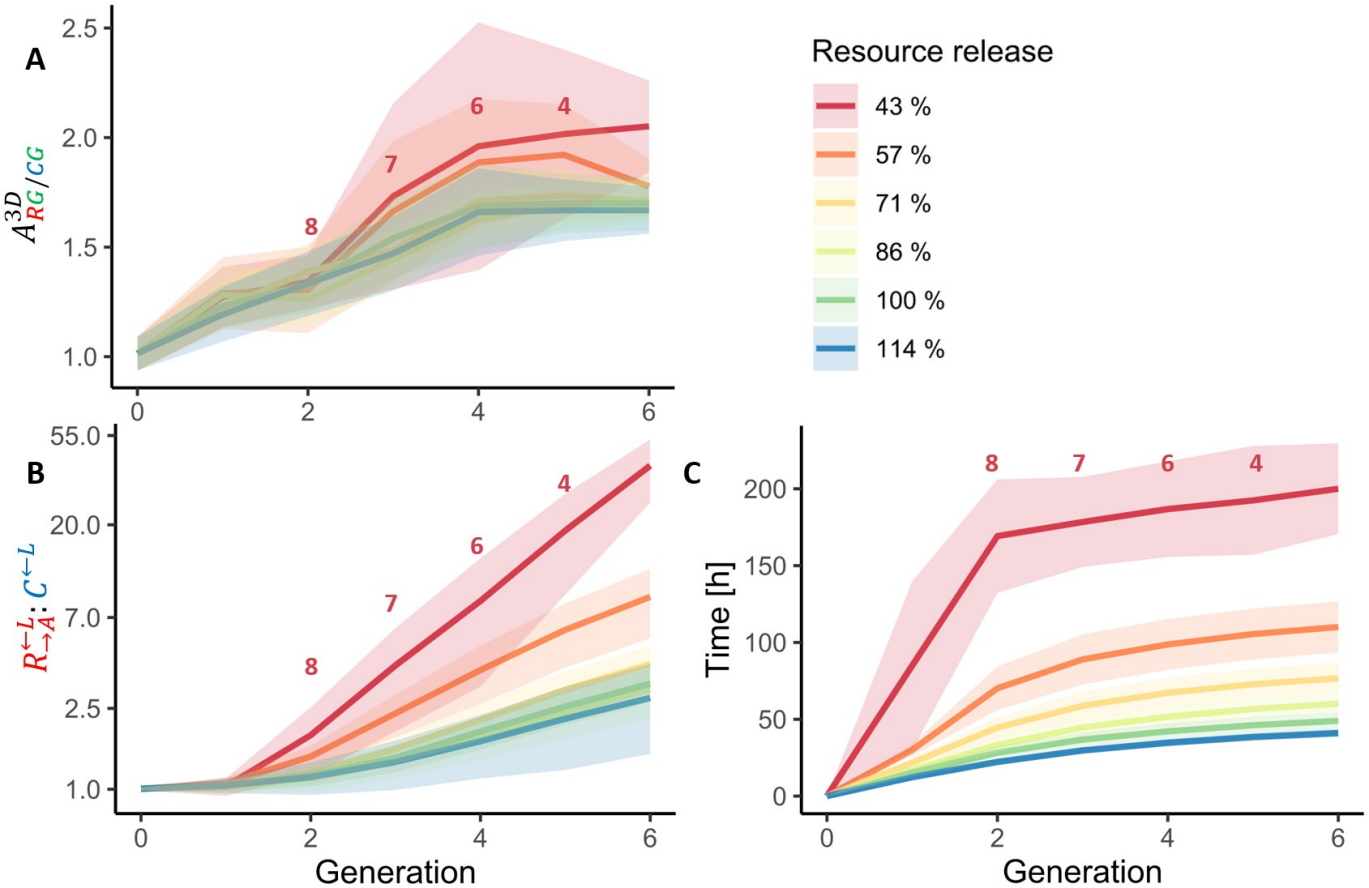
Reduced resource release rates increase abundance and intermixing of cooperators but also their generation time. Simulations were performed with *McComedy* with varied resource release rates and all other parameter values corresponding to scenario without supplemented lysine and adenine (- LA) in Fig 5. At low resource release rates, not all simulated communities achieved six generations. Numbers indicate how many of the initial 10 simulations contributed to the data visualized in the same color, starting from the respective X-position. Ribbons indicate the standard deviation. **A**: Association index of the two cooperative strains ($R_{\to A}^{\leftarrow L}$ with $G_{\to L}^{\leftarrow A}$) and the non-cooperators **C**^**←L**^. **B**: Abundance ratio between the cooperators $R_{\to A}^{\leftarrow L}$ and the non-cooperators **C**^**←L**^. **C**: Mean time until respective generation time is reached. One generation corresponds to the biomass doubling time of the simulated community.

According to Momeni *et al*. [16], the intermixing of two cooperative strains depends on the amount of the essential resources that is exchanged between strains. This means that if a cooperative strain reduces the release of the shared resource, it will also intermix less with its cooperation partners and, thus, be inferior to another, more cooperative strain, even though it saves some of the cost for producing the cooperative benefit [16]. This finding raises a follow-up question: How would the system behave if both genotypes $R_{\to A}^{\leftarrow L}$ and $G_{\to L}^{\leftarrow A}$ would simultaneously exhibit an increased or reduced cooperativity? Using *McComedy*, we examined this situation. A reduced overall cooperativity in terms of resource release by both cooperative strains led to an increased intermixing and relative abundance of cooperators (Fig 6), which might seem counterintuitive. However, a reduced resource release results in less resources that diffuse in the environment. Thus, resources are mostly available in short distances to the respective producing (cooperative) individuals, which leads to a stronger localization of cooperative benefit, thus favoring intermixing as discussed by Momeni *et al*. [16]. Note that this strong spatial intermixing due to reduced cooperation coincided with considerably slower growth of the entire population (i.e. longer generation times, Fig 6C). For very low rates of resource release, cooperators were not able to sustain the whole population, resulting in extinction after few generations. Therefore, the question arises, whether the model system is evolutionary unstable, albeit robust against non-cooperators in the short term. This example shows that the model implemented with *McComedy* serves as a powerful tool to understand mechanisms that drive spatial self-organization of microorganisms and also hints to possible challenges when evolutionary dynamics are taken into account.

## Discussion

In this study, we introduced *McComedy*, a tool for developing and analyzing next-generation IBMs of microbial consumer-resource systems. The goal was to create a modeling framework that (i) allows to accurately capture the spatiotemporal dynamics of microbial communities as a consequence of mechanistically sound simulations of the relevant processes, (ii) is highly modularized to facilitate simulation of various distinct microbial systems by combining the relevant processes, and (iii) is user-friendly and accessible to researchers without profound programming knowledge. *McComedy* was successfully tested by reproducing the experimental and model results of two published studies that analyzed the spatiotemporal dynamics of microbial communities with consumer-resource interactions. In both cases, *McComedy* was additionally applied to investigate the studied systems beyond the scope of the original publications, providing additional insights that complement the original studies. Thus, it was also demonstrated how *McComedy* can be flexibly adjusted to assess new scenarios.

We consider the good correspondence of our model results with previously published data a consequence of *McComedy*’s generic process modules that were combined and interact in the specific IBMs. Such simulation of standardized low-level processes and their interplay favors the emergence of structural realism, which means that a model largely captures the functional and organizational structure of a system [24]. As the two IBMs that we implemented build on similar sets of process modules to be combined (Table 1) and adhere to *McComedy*’s fundamental design concepts and assumptions (e.g. continuous spatial positions of the microorganism individuals), they are very similar with respect to the model structure and implementation details. In contrast, the two IBMs provided by Mitri *et al*. [17] and Momeni *et al*. [16] are designed quite differently. For example, the former model is based on explicit individuals representing microorganisms, whereas the latter model is based on local densities of microorganisms in discrete spatial grid cells. While both models accurately represent the respective microbial system, an attempt to compare or synthesize the results of the two studies would be hampered by the different designs used. This raises the question of how much of the different observations can be attributed to distinct ecological processes and how much is a consequence of the different design choices. By simulating both scenarios with *McComedy*, we firstly corroborate the generality of the respective findings and secondly demonstrate how the approach of building IBMs from generic process modules contributes to a coherent understanding of different ecological processes.

**Table 1:** Process modules currently available in *McComedy*. The columns SOM (Spatial organization model, Mitri *et al*. [17]) and CM (Cooperation model, Momeni *et al*. [16]) indicate with an ‘X’ which process modules were integrated in the corresponding *McComedy* models. A more detailed description of each process module is provided in the ODD protocol (S1 Text).

Process module	Description	SOM	CM
*Attachment*	Upon physical contact, microbes can attach to each other.		
*CellPartition*	This module estimates the overlap of each microbial cell with resource grid cells. It is required by some other processes and enhances the computation time of the simulation.	**X**	**X**
*ChangeGenotype*	Microbes change their type with a predefined probability.		**X**
*ConstantProduction*	Microbes produce a resource at a predefined constant rate. The resource remains in an intracellular pool.		**X**
*ConstantResourceBoundaries*	Resource concentrations are held constant at the boundaries of the environment (opposed to default periodic boundary conditions).	**X**	**X**
*Diffusion*	Resources diffuse through the environment at a predefined rate.	**X**	**X**
*Flow*	Microbes move in a predefined direction. This does not affect resources.		**X**
*Growth*	Consumed resources that have been allocated for growth are transformed into biomass with a predefined yield.	**X**	**X**
*ImpermeableMicrobeBoundaries*	Microbes cannot penetrate the boundaries of the system (opposed to default periodic boundary conditions).		**X**
*InitBiofilm*	Upon simulation start, microbes are placed on a two-dimensional plane at the bottom of the simulated environment.		**X**
*InitCluster*	Upon simulation start, microbes are distributed within a sphere at the center of the simulated environment.	**X**	
*InitModel*	This is the only obligatory process module. It attaches the initial resources and microbes to the environment and sets the initial values of the state variables.	**X**	**X**
*LocalSource*	Resource concentrations are increased or reduced at one or multiple locations according to a predefined rate.		
*Lysis*	Microbes are removed from the environment at a given probability.		
*PassiveRelease*	Microbes release produced intracellular resources into resource grid cells that the microbes overlap with.		**X**
*PassiveUptake*	Microbes consume resources from grid cells that the microbes overlap with, according to Monod-kinetics.	**X**	**X**
*ProximityManager*	This module groups microbes that are close to each other in order to boost searching algorithms. It is required by some other processes and enhances the computation time of the simulation.	**X**	**X**
*Replication*	When a microbe’s biomass exceeds a predefined value, it is divided into two individuals.	**X**	**X**
*ResourceDecay*	At all grid positions, the concentration of resources decays at predefined rates.		
*Shoving*	Microbes that overlap spatially push each other away.	**X**	**X**
*Starving*	Microbes that are marked as starving are removed from the environment.		
*SubstrateUtilization*	Intracellular resources are reduced at a predefined rate to account for maintenance costs. The remainder is allocated to biomass growth. If the maintenance cost exceeds the amount of intracellular resources, the microbe is marked as starving.	**X**	**X**

In terms of providing a framework for IBMs of microbial communities, *McComedy* is not the first of its kind. There are several other prominent and highly useful examples like Simbiotics [33], iDynoMiCS [28], NUFEB [30], COMETS [26], and Biocellion [34]. Also NetLogo [35] is a versatile and widely used framework for individual-based modeling of, for instance, microbial communities [36,37]. Although tailored for a broader community, these frameworks can be challenging and time-consuming to master for non-experts. Therefore, *McComedy* can be particularly useful to microbial ecologists and modelers who have little experience with programming and cannot invest much time into learning the specifics of other frameworks. This also offers the possibility of using *McComedy* for teaching purposes. The intuitive user interface and high flexibility allow an easy entry into individual-based modeling. In this context, student projects could for example constitute the reproduction of existing studies, as presented in this work.

With *McComedy*, the output data of the IBMs allows for sophisticated analysis of the simulated community dynamics across spatial scales and organizational levels. For example, simulation data on the biomass of each individual microbe over time can be aggregated into population dynamics (Figs 5C, 5D, 6B and 6C) and data on the spatial position of each individual microbe can be used for spatial pattern analysis (Figs 2D, 3B, 5A, 5B and 6A). The possibility to analyze the microbial communities across spatial scales and organizational levels allows for pattern-oriented modeling, a technique where the model output is matched with as many different empirical patterns as possible to increase structural realism and reduce complexity [38].

Current limitations for a broad application of *McComedy* for the modeling of various microbial systems are given by the set of available process modules. With the presented version of *McComedy*, we provide a library of selected process modules that allow for microbial community modeling with a focus on spatially explicit interactions and consumer-resource dynamics. At the current stage, *McComedy* facilitates modeling communities that consist of sessile microorganisms (e.g. in colonies and biofilms), planktonic individuals that move randomly in a liquid medium, and microbial aggregates suspended in liquid medium.

Process modules encompass diffusion and decay of resources, passive movement, attachment, and shoving of microbes, different initial microbe distributions, boundary conditions, a simple metabolism of microbes that optionally involves production and/or consumption of resources, and microbial growth and replication. However, there are additional processes that might be relevant in microbial communities but are not yet covered by the currently available catalogue of process modules. Therefore, we will continue the development of *McComedy* and provide more process modules in future versions that will allow for a wider range of microbial IBMs. For example, we are currently working on other forms of metabolic interactions such as direct resource exchange via nanotubes [39,40] and evolutionary mechanisms such as mutation of microbial traits. Furthermore, future versions of *McComedy* will facilitate active microbial movement (e.g. based on chemotaxis) and negative metabolic interactions (e.g. release of growth-inhibitory by-products). Due to the free access to the code of *McComedy*, further process modules could be developed by other modelers too, if they are proficient with the programming language Java.

Another limitation of *McComedy* concerns the size of both the environment and the microbial communities that can be simulated. Although, technically, there are no hard limits for either of these, we recommend to not exceed community sizes of 2,000 individuals or environments of 50,000 grid cells to ensure reasonable computation times (see also computation times for representative simulations in S2 File). This recommended scale allows to analyze the community dynamics at the level of individual cells, which is the intended use of *McComedy*. Larger-scale simulations, for example on the scale of an entire test tube, should be rather conducted on a more aggregated level using other modeling frameworks.

Based on the successful testing of *McComedy* with different studies from the literature and due to the simple and fast model creation, we conclude that *McComedy* is a promising tool for users who require next-generation IBMs of spatially explicit microbial consumer-resource systems. As shown in this work, the flexibility to model different systems does not come at the cost of accuracy because the system dynamics emerge from the mechanistic interplay of the generic process modules, close to what happens in the real systems. The development of *McComedy* goes on and we invite all researches from fields related to microbial ecology to try applying *McComedy* within their own projects.

## Methods

### Implementation of *McComedy*

In this section, an overview over the implementation of *McComedy* is provided. For a complete description and implementation details, refer to the ODD (overview, design concepts and details) protocol for standardized descriptions of individual-based models [41,42] (S1 Text). *McComedy* is implemented in Java 11 using the JavaFX library for the graphical user interface. The process modules, each represented by one Java class, have been tested with the JUnit unit-testing framework. *McComedy* is open-source and the code can be viewed and downloaded from https://git.ufz.de/bogdanow/mccomedy.

A microbial community modeled with *McComedy* is represented by a spatially explicit three-dimensional environment in which resources have local concentrations in discrete grid cells and microbe entities with a spherical shape have continuous position coordinates. At each time step the state of the system is defined by a set of state variables that are attached to the environment and individual microbes. The list of state variables is provided in the section *2*. *Entities*, *state variables*, *and scales* of the ODD protocol (S1 Text).

Over simulated time, the state variables are subject to change by a set of process modules. The process modules are repeatedly executed at a specific frequency and simulate natural processes, which are assumed to shape the dynamics of the modeled system (Table 1). Please see section *7*. *Submodels* of the ODD protocol (S1 Text) for detailed descriptions. As the simulation runs, the state of the system (i.e. values of all state variables) is saved in model output files in predefined intervals. This enables analyses of the temporal progression of the modeled system.

### Spatial organization model (Mitri *et al*. [17])

Corresponding to the original model by Mitri *et al*. [17], the system was simulated in an approximately two-dimensional environment of size 250 μm x 250 μm x 1 μm. The model was initialized with one homogeneously distributed resource *R* and two types of microbes, *M1* and *M2*, both of which could consume *R* and were also identical in all other respects except for the name and color. From each type, 100 microbes were randomly placed in a cluster at the center of the simulated environment (process module *InitCluster*).

The process modules *PassiveUptake*, *SubstrateUtilization*, and *Growth* were integrated into the model to account for resource uptake and biomass growth according to Monod-kinetics [43]. Microbes were assumed to divide into two individuals upon exceeding a critical biomass (process module *Replication*). Mechanical interaction between microbes (i.e. pushing each other away when overlapping spatially) was simulated by the process module *Shoving* according to the algorithm described in [28]. The process module *Diffusion* was used to simulate resource diffusion throughout the environment. The ‘agar plate’ that contained the resources and on which the microbes grew was assumed to extend far beyond the simulation boundaries. Therefore, resource concentrations at the boundaries of the simulated environment were maintained at the initial resource concentration to account for diffusion into the simulated system (process module *ConstantResourceBoundaries*).

Across different simulations, the parameter values for the initial resource concentration and diffusion constant were varied. Five replicates for each variant of parametrization were simulated, each of which differed in the initial distribution of microbes due to different random generator seeds. However, the replicates for different variants of parametrization were initialized and simulated with the same random generator seeds. The generic units of *McComedy* for time T, distance S, resource mass M, and microbial dry mass M* were treated as seconds, micrometers, femtograms, and femtograms (dry weight), respectively. A complete list of model parameters and their values is provided in S1 File.

Simulations were set to run for a maximum of 100 hours or until the community reached a total abundance of 20,000 microbes. The statistical analysis was conducted at the time point, at which the first simulation stopped, which was the case after 45 simulated hours, when initial concentrations were varied and after 39 hours, when the diffusion constant was varied.

Top-views on colonies (Figs 2C and 3A) were rendered with *McComedy*. The quantitative analysis of the heterozygosity and the demixing distances was performed according to Mitri *et al*. [17]. The heterozygosity was calculated by sampling boxes of 5 μm x 5 μm along transects from the initial inoculum to the edge of the colony and counting individuals of *M1* and *M2* in each box. The heterozygosity as a function of distance from the inoculum is given by

$$H\left(x\right)=\frac{2}{\Phi }\sum_{\varphi }^{\Phi }f_{1}\left(x,\;\varphi \right)(1-f_{1}(x,\varphi )),$$

Where *f*_1_(*x*, *φ*) is the proportion of microbes *M1* at distance *x* from the inoculum location in transect *φ* and Φ is the number of transects. The demixing distance is defined as the point where $\frac{dH}{dx}$, the derivative of the heterozygosity function, is minimal.

### Cooperation model (Momeni *et al*. [16])

The microbial system was simulated in a three-dimensional environment of size 480 μm x 100 μm x 240 μm. Two resources, *L* and *A* (representing lysine and adenine, respectively) and three types of microbes,$R_{\to A}^{\leftarrow L}$, $G_{\to L}^{\leftarrow A}$, and *C*^←*L*^, were added to the environment. The model was initialized with either empty resource grid cells for the scenario in which lysine and adenine were not provided (*–LA*) or with inexhaustibly high resource concentrations (i.e. 9999999 fmole/125 μm^3^) for the scenario in which lysine and adenine were provided (*+ LA*). Initially, 115 microbes of each type were randomly distributed on a two-dimensional plane (orthogonal to the Y-axis) close to the bottom of the simulated environment (process module *InitBiofilm*). This plane represented the surface of the agar, on which microbes were growing. Note that in the original study [16], vertical positions are described by Z-coordinates, whereas in *McComedy*, vertical positions are described by Y-coordinates.

Microbes were restricted from movement below the surface of the agar by the process module *ImpermeableMicrobeBoundaries* and a weak gravitational force was simulated by moving the microbes towards the agar surface with the process module *Flow*. As in the previous example, resource consumption and metabolism were modeled with the process modules *PassiveUptake*, *SubstrateUtilization*, and *Growth*. Additionally, resource overproduction and release were integrated with the process modules *ConstantProduction*, *PassiveRelease*, *and ChangeGenotype*. To account for mortality, the strain R, which constantly produced adenine, changed with a low probability to a metabolically inactive type. Strain G, which released lysine only upon cell death, was modeled such that it did not produce lysine when active. In the case of mortality (also occurring with a low probability) it first changed to a temporary type that produced a high amount of lysine and after one more time step to a metabolically inactive type. The process module *Diffusion* was used to simulate resource diffusion throughout the environment. The boundaries in X- and Z-direction were kept periodic (*McComedy* default) and resource concentrations at Y-boundaries were maintained at the initial concentration to simulate open boundaries (process module *ConstantResourceBoundaries*).

10 replicates were simulated for every variant of parametrization, each of which differed in the initial distribution of microbes due to different random generator seeds. However, the replicates for different variants of parametrization were initialized and simulated with the same random generator seeds. The generic units of *McComedy* for time T, distance S, resource mass M, and microbial dry mass M* were treated as seconds, 5 micrometers, femtomoles, and 10 picograms (dry weight), respectively. A complete list of model parameters and their values is provided in S1 File.

The simulations ran until the community reached a total abundance of 22,080 microbes (i.e. 6 doublings of the initial 345 microbes) or until all microbes were dead. The analyses were performed at all time points at which the community size doubled (i.e. when the community abundance was closest to 345; 690; 1,380; 2,760; 5,520; 11,040; and 28,080 microbes, respectively).

Vertical cross-section views (Fig 4E and 4F) were rendered with *McComedy*. The ratio between two types was calculated with respect to the biomass of each type. The association index is given by

$$A=\frac{\frac{1}{n_{{\;R}_{\to A}^{\leftarrow L}}}\sum_{i}^{n_{Y1}}a({{\;R}_{\to A}^{\leftarrow L}}_{i},G_{\to L}^{\leftarrow A})}{\frac{1}{n_{C^{\leftarrow L}}}\sum_{j}^{n_{Y3}}a({C_{}^{\leftarrow L}}_{j},\;G_{\to L}^{\leftarrow A})},$$

where $n_{R_{\to A}^{\leftarrow L}}$ is the number of microbes of type $R_{\to A}^{\leftarrow L}$ that are in proximity of at least one microbe of a different type and $a({R_{\to A}^{\leftarrow L}}_{i},G_{\to L}^{\leftarrow A})$ is the number of microbes of type $G_{\to L}^{\leftarrow A}$ that are in proximity of the *i*-th microbe of type $R_{\to A}^{\leftarrow L}$ (microbes of type $R_{\to A}^{\leftarrow L}$ that have no microbes of a different type in their proximity are excluded). Here, ‘*in proximity*’ means a maximum distance of 7.5 μm between the midpoints of the two microbes, which includes almost only directly adjacent microbes. The variables in the denominator are defined analogously.

### Statistical analysis

Statistical analysis was conducted with R 4.0.3 [44]. Plots were created with the package ‘ggplot2’ [45]. R-scripts are provided in S1 Scripts.

## Supporting information

S1 File Processes and parameters. (PDF)

S2 File Computational performance. (PDF)

S1 Text McComedy ODD protocol. (PDF)

S1 Scripts R-scripts for data analysis. (ZIP)

S2 Scripts R-scripts for data pre-processing. (ZIP)
