# Supplementary material for: McComedy: A user-friendly tool for next-generation individual-based modeling of microbial consumer-resource systems
Source: PLoS Comput Biol. 2022 Jan 24;18(1):e1009777. doi: 10.1371/journal.pcbi.1009777 (PMC8830788; doi:10.1371/journal.pcbi.1009777)
Supplement: S1 File — (PDF) [file pcbi.1009777.s001.pdf]

Processes and parameters

Spatial organization model (Mitri *et al.* 2015 [1])

Table 1. Process modules

**Process**	**Time step [ms]**
CellPartition	1000
ConstantResourceBoundaries	100
Diffusion	100
Growth	1000
InitCluster	-
InitModel	-
PassiveUptake	1000
ProximityManager	1000
Replication	1000
Shoving	10000
SubstrateUtilization	1000

Table 2. Resource parameters

**Parameter**	**Value for resource R**	**Reference**
resource name	'R'	Model assumption
initial concentration [fg/µm³]	20, 40, 60, 80, 100, 120, 140, 160 (120 when diffusion constant was varied)	The range of variation adapted from [1]. Absolute values set such that colonies grew within two days
diffusion constant [µm²/s]	0.015, 0.035, 0.055, 0.075, 0.095 (0.055 when initial concentration was varied)	Model assumption
constant X-boundary	TRUE	Model assumption
constant Y-boundary	TRUE	Model assumption
constant Z-boundary	FALSE	Model assumption
concentration at boundary [fg/µm³]	'As initial concentration'	Model assumption

Table 3. Microbe parameters

**Parameter**	**Values for type M1**	**Values for type M2**	**Reference**
genotype	‘M1’	‘M2’	Model assumption
initial abundance	100	100	Adapted from [1]
biomass density [fg/μm^3]	375	375	Estimated from [2]
min biomass [fg]	250	250	Estimated from [2]
max biomass [fg]	500	500	Estimated from [2]
consumes resource	R	R	Adapted from [1]
maintenance cost [1/s]	0	0	Model assumption
half-saturation constant [fg/μm^3]	1.5	1.5	Adapted from [1]
max uptake [1/s]	0.00039	0.00039	Adapted from [1]
yield rate	0.5	0.5	Adapted from [1]

Table 4. Model parameters

**Parameter**	**Value**	**Reference**
spatial extent X [µm]	250	Model assumption
spatial extent Y [µm]	250	Model assumption
spatial extent Z [µm]	1	Model assumption
simulation time [s]	360000	-
max microbes number	20000	-
stop when all microbes die	TRUE	-
constant initial position	FALSE	-
random generator seed	1	-
proximity raster cell size [µm]	3	Model assumption

Cooperation model (Momeni *et al.* 2013 [3])

Table 5. Process modules

**Process**	**time step [ms]**
CellPartition	10000
ChangeGenotyoe	100
ConstantProduction	100
ConstantResourceBoundaries	100
Diffusion	100
Flow	10000
Growth	10000
ImpermeableMicrobeBoundaries	10000
InitBiofilm	-
InitModel	-
PassiveRelease	100
PassiveUptake	100
ProximityManager	10000
Replication	10000
Shoving	10000
SubstrateUtilization	10000

Table 6. Resource parameters

**Parameter**	**Value for Resource L**	**Value for Resource A**	**Reference**
resource name	'L'	'A'	Adapted from [3]
initial concentration [fmole/(5 µm)³]	0, 9999999 (0 when release rate was varied)	0, 9999999 (0 when release rate was varied)	Model assumption
diffusion constant [(5 µm)²/s]	0.01	0.01	Model assumption
constant X-boundary	FALSE	FALSE	Model assumption
constant Y-boundary	TRUE	TRUE	Model assumption
constant Z-boundary	FALSE	FALSE	Model assumption
concentration at boundary [fmole/(5 µm)³]	As initial resource concentration	As initial resource concentration	Model assumption

 Table 7. Microbe parameters (living microbes and temporary type of $G_{\rightarrow L}^{\leftarrow A}$)

**Parameter**	**Value for type $R_{\rightarrow A}^{\leftarrow L}$**	**Value for type $G_{\rightarrow L}^{\leftarrow A}$**	**Value for type $G_{\rightarrow L}^{\leftarrow A}(dying)$**	**Value for type $C^{\leftarrow L}$**	**Reference**
genotype	'R'	'G'	'GX'	'C'	Adapted from [3]
initial abundance	115	115	0	115	Adapted from [3]
biomass density [fmole/(5 µm)³]	4.688	4.688	4.688	4.688	Estimated from [4]
min biomass [10 pg]	1.1	1.1	0	1.1	Estimated from [4]
max biomass [10 pg]	2.2	2.2	9999	2.2	Estimated from [4]
consumes resource	L	A	-	L	Adapted from [3]
releases resource	A	-	L	-	Adapted from [3]
maintenance cost [fmole/10 pg/s]	0	0	-	0	Model assumption
half-saturation constant [fmole/(5 µm)³]	0.00013	0.00013	-	0.00013	Adapted from [3]
max uptake [fmole/10 pg/s]	0.000018	0.000018	-	0.000018	Calculated from minimal doubling time [3] and yield rate
release rate [fmole/10 pg/s]	0.000003, 0.000004, 0.000005, 0.000006, 0.000007, 0.000008 (0.000007 when initial concentration was varied)	0.000003, 0.000004, 0.000005, 0.000006, 0.000007, 0.000008 (0.000007 when initial concentration was varied)	1.5, 2, 2.5, 3, 3.5, 4 (3.5 when initial concentration was varied)	0.000003, 0.000004, 0.000005, 0.000006, 0.000007, 0.000008 (0.000007 when initial concentration was varied)	Model assumption
yield rate [10 pg/fmole]	10	10	-	10.2	Model assumption. Fitness advantage of $C^{\leftarrow L}$ adapted from [3]

impermeable X-boundaries	FALSE	FALSE	FALSE	FALSE	Model assumption
impermeable Y-boundaries	TRUE	TRUE	TRUE	TRUE	Model assumption
impermeable Z-boundaries	FALSE	FALSE	FALSE	FALSE	Model assumption
impermeable boundary offset [5 µm]	3	3	3	3	Model assumption
change genotype to	D1	GX	D2	D3	Model assumption
change genotype probability [1/s]	0.000015	0.000005	1	0.000015	Adapted from [3]

Table 8. Microbe parameters (dummies, representing dead cells)

**Parameter**	**Value for type D1**	**Value for type D2**	**Value for type D3**	**Reference**
genotype	'D1'	'D2'	'D3'	Model assumption
initial abundance	0	0	0	Adapted from [3]
biomass density [fmole/(5 µm)³]	4.688	4.688	4.688	Estimated from [4]
min biomass [fmole]	0	0	0	Model assumption
max biomass [fmole]	9999	9999	9999	Model assumption
consumes resource	-	-	-	Model assumption
releases resource	-	-	-	Model assumption
maintenance cost [fmole/10 pg/s]	-	-	-	Model assumption
half-saturation constant [fmole/(5 µm)³]	-	-	-	Model assumption
max uptake [fmole/10 pg/s]	-	-	-	Model assumption
release rate [fmole/10 pg/s]	-	-	-	Model assumption
yield rate [10 pg/fmole]	-	-	-	Model assumption
impermeable X-boundaries	FALSE	FALSE	FALSE	Model assumption
impermeable Y-boundaries	TRUE	TRUE	TRUE	Model assumption
impermeable Z-boundaries	FALSE	FALSE	FALSE	Model assumption

impermeable boundary offset [5 µm]	3	3	3	Model assumption
change genotype to	-	-	-	Model assumption
change genotype probability [1/s]	-	-	-	Model assumption

Table 9. Model parameters

**Parameter**	**Value**	**Reference**
spatial extent X [5 µm]	96	Model assumption
spatial extent Y [5 µm]	20	Model assumption
spatial extent Z [5 µm]	48	Model assumption
simulation time [s]	864000	-
max microbes number	22080	-
stop when all microbes die	TRUE	-
constant initial position	FALSE	-
random generator seed	1	-
proximity raster cell size [5 µm]	1	Model assumption
biofilm Y-position [5 µm]	3	Model assumption
mean flow X [5 µm/s]	0	Model assumption
mean flow Y [5 µm/s]	-0.001	Model assumption
mean flow Z [5 µm/s]	0	Model assumption
flow SD X [5 µm/s]	0	Model assumption
flow SD Y [5 µm/s]	0	Model assumption
flow SD Z [5 µm/s]	0	Model assumption

References

1. Mitri S, Clarke E, Foster KR. Resource limitation drives spatial organization in microbial groups. The ISME Journal. 2015;10(6): 1471-82. doi: 10.1038/ismej.2015.208.
2. Neidhardt FC, Umbarger HE. Escherichia coli and Salmonella: cellular and molecular biology. 2 ed. Washington, D.C.: ASM Press; 1996.
3. Momeni B, Waite AJ, Shou W. Spatial self-organization favors heterotypic cooperation over cheating. Elife. 2013;2: e00960. doi: 10.7554/eLife.00960.
4. Klis FM, de Koster CG, Brul S. Cell wall-related bionumbers and bioestimates of Saccharomyces cerevisiae and Candida albicans. Eukaryotic Cell. 2014;13(1): 2-9. doi: 10.1128/EC.00250-13.
