# Supplementary material for: McComedy: A user-friendly tool for next-generation individual-based modeling of microbial consumer-resource systems
Source: PLoS Comput Biol. 2022 Jan 24;18(1):e1009777. doi: 10.1371/journal.pcbi.1009777 (PMC8830788; doi:10.1371/journal.pcbi.1009777)
Supplement: S2 File — (PDF) [file pcbi.1009777.s002.pdf]

Computational performance

We measured the computation time for a set of representative simulations. The time step length, microbial abundance, and spatial extent of the simulated environment were varied. The simulations were performed on an ordinary laptop. The following tables specify the parametrization of the simulations (Tables 1-5) and provide details concerning the hardware of the laptop (Table 6).

Processes and parameters

Table 1. Process modules

**Process**	**Time step [ms]**
CellPartition	100, 1000
Diffusion	100, 1000
InitModel	-
PassiveUptake	100, 1000
ProximityManager	100, 1000
Growth	100, 1000
Shoving	100, 1000
SubstrateUtilization	100, 1000
Flow	100, 1000

Table 2. Resource parameters

**Parameter**	**Value for resource R1**
resource name	‘R1’
initial concentration [M/V]	999999
diffusion constant [S^2/T]	1
resource color hue (0 to 1)	0.6
max render concentration [M/V]	1

Table 3. Microbe parameters

**Parameter**	**Values for type M1**
genotype	‘EC1’
initial abundance	10, 100, 1000, 10000
biomass density [M*/V]	375
min biomass [M*]	250
max biomass [M*]	500
consumes resource	‘R1’
maintenance cost [1/s]	0
half-saturation constant [fg/μm³]	0.0001
max uptake [1/s]	0.00000186
yield rate	136.3
microbe color hue [0 to 1]	0

Table 4. Model parameters

Parameter	Value
spatial extent X [S]	25, 50, 75, 100
spatial extent Y [S]	25
spatial extent Z [S]	25
simulation time [T]	120
max microbes number	99999
mean flow X [S/T]	0
mean flow Y [S/T]	0
mean flow Z [S/T]	0
flow SD X [S/T]	0.1
flow SD Y [S/T]	0.1
flow SD Z [S/T]	0.1
stop when all microbes die	TRUE
constant initial position	FALSE
random generator seed	1
proximity raster cell size [µm]	3

Table 5. Settings

Parameter	Value
replicates	1
result directory name	Results
save microbe data every [T]	0
save resource data every [T]	0
save microbe image every [T]	0
save resource images every [T]	0
log frequency [mT]	5000
draw in 3D	TRUE
simultaneous runs	1

Hardware

Table 6. Test-simulation executed on following hardware

Device	Notebook Lenovo T480
CPU	Intel 8th Gen Quad-Core i5-8250U
CPU speed	1.6 GHz
Memory	16 GB DDR4 RAM

Performance

The duration to simulate one virtual second was assessed for different parametrizations (Figs 1 and 2). Overall the computation time increased with higher microbe numbers, larger environment size, and shorter time steps (ΔT). However, at very high microbe numbers, reducing the environment sizes (i.e. increasing the density) resulted in increased computation time as the computationally expensive *Shoving* algorithm had to shift more microbes.

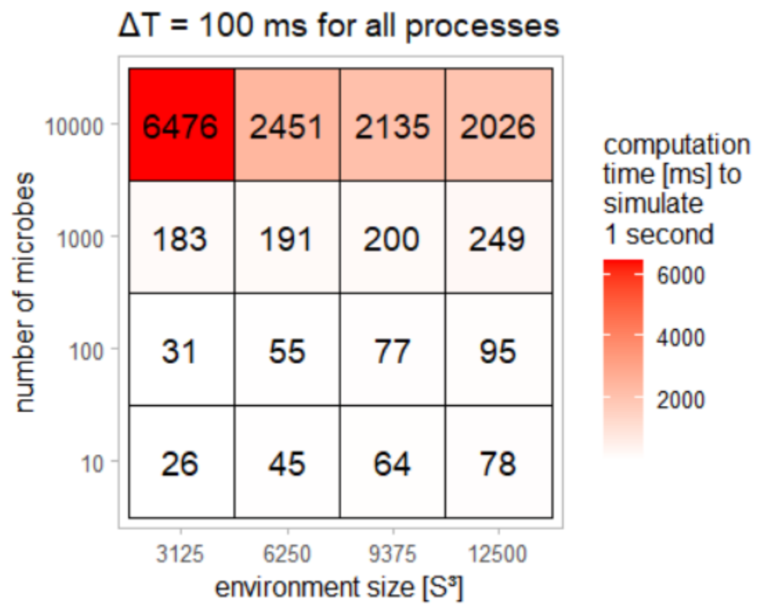

Fig 1. Computation time for ΔT = 100 ms for all processes.

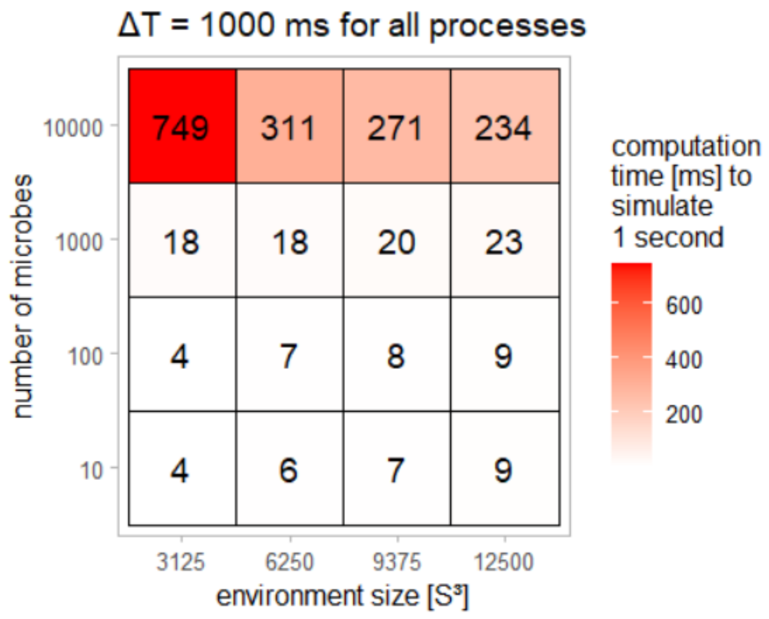

Fig 2. Computation time for ΔT = 1000 ms for all processes.
