# Supplementary material for: McComedy: A user-friendly tool for next-generation individual-based modeling of microbial consumer-resource systems
Source: PLoS Comput Biol. 2022 Jan 24;18(1):e1009777. doi: 10.1371/journal.pcbi.1009777 (PMC8830788; doi:10.1371/journal.pcbi.1009777)
Supplement: S1 Text — (PDF) [file pcbi.1009777.s003.pdf]

McComedy ODD Protocol

The model description follows the ODD (Overview, Design concepts, Details) protocol for describing individual- and agent-based models [1,2]. Note that the ODD protocol was developed for specific models whereas McComedy is a generic modeling platform. Hence, where the ODD protocol requires particular information with regard to single models, possibilities in different implementations of specific models using the McComedy platform are described.

1. Purpose and patterns

The purpose of McComedy is to provide a platform for spatially explicit individual-based models (IBMs) of microbial communities including metabolic interactions. The platform allows for simple and fast model creation by selecting a set of process modules to be combined in one specific model. The IBMs created with McComedy can serve different purposes, however, they have in common that they simulate the spatiotemporal dynamics of microbial communities driven by metabolic processes. Therefore, they allow for mechanistic analyses and understanding of these dynamics. In this manner, specific IBMs may be used to recapitulate and explain empirical findings, to study parameter setups that cannot (easily) be controlled for in experiments, and to generate new hypotheses on the mechanisms underlying community dynamics.

The accuracy of the models can be assessed by comparison of emerging patterns in the model and corresponding experiments, provided that such data exists. Such patterns can be, for example, growth dynamics over time, relative abundances of different types of microbes, and spatial organization as quantifiable by, for example, intermixing and cluster analysis.

2. Entities, state variables, and scales

The entities in McComedy are microbes and the environment. Microbes are the individuals that constitute the investigated communities. They can, for example, represent bacteria, fungi, or protists. These microbes have a spherical shape. Each individual is characterized by several state variables such as its genotype, biomass or spatial position (see Table 1 for all state variables). The individuals' size is determined by their biomass and a density parameter. Certain microbe state variables are included only when the corresponding process module is used (Table 1).

The environment is a three-dimensional array of discrete grid cells. It contains the resources as well as the individuals. The individuals have exact continuous spatial positions, meaning that they can overlap with more than one grid cell (Table 1). Different types of resources represent different metabolites which are consumed or produced by the microbes. Resource concentrations are assigned to discrete spatial grid cells. Hence, the state variables characterizing the environmental grid cells are the local resource concentrations of the different types of resources (Table 1).

Table 1. State variables in McComedy. The symbols are used for the state variables in formulas in this ODD protocol, but not in the source code of McComedy. The column "Process module" indicates if a state variable is only included when the specified process module is used.

**Name**	**Symbol**	**Variable type**	**Explanation**	**Process module**
ID	-	Integer	A unique ID which allows tracking an individual microbe over simulation time.	-
Genotype	-	String	Defines the type of a microbe	-
Biomass	B	Decimal	A microbes dry weight	-
X-position	X	Decimal	X-position of a microbe's center	-
Y-position	Y	Decimal	Y-position of a microbe's center	-
Z-position	Z	Decimal	Z-position of a microbe's center	-
Local concentrations	C_R	3D-Array of Decimals	The concentration of a resource R in each grid cell of the discretized simulated space	-
Attached microbes	-	List of Microbes	Lists all microbes that are currently attached to a microbe	*Attachment*
Substrate pool	S	Decimal	Intracellular pool of resources in a microbe that have been consumed but not utilized yet	*SubstrateUtilization*
Starving	-	Boolean	Indicates whether a microbe is starving	*SubstrateUtilization*
Growth resources	G	Decimal	Resources allocated to biomass growth in a microbe	*Growth*
Product pool	P_R	Decimal	Intracellular pool of resource R that have been produced by a microbe	*ConstantProduction*

The model acts on the scale of individual microbes, simulating up to several thousand individuals. McComedy works with generic units for time (T), distance (S), resource mass (M) and microbial dry mass (M*). The modeler is required to decide in which specific units the generic ones translate and parametrize the model accordingly. For example, assuming that T corresponds to 1 s and S corresponds to either 1 µm or 5 µm, a *diffusion constant* of 25 $\mu m^2/s$ needs to be defined as either 25 S^2/T or 1 S^2/T, respectively. For best computational performance, S should be in the same order of magnitude as a typical microbe's diameter. For instance, when modeling the bacteria *Escherichia coli*, S could be considered 1 µm. The extent of the environment and the number of microbes are only restricted by the computational time needed to simulate all processes. We suggest starting with a spatial extent of 125 x 125 x 1 S^3 (2D) or 25 x 25 x 25 S^3 (3D) and a maximum microbe number of 2000 (these number can be increased when simulating on high-performance computing systems).

3. Process overview and scheduling

Processes are the building blocks of every McComedy model. The processes are encapsulated into mostly independent *process modules*, which can be combined in several ways, resulting in a high flexibility in model design. To this end, the graphical user interface of McComedy allows selecting from a list of all available process modules. Restrictions are that some process modules require others to be included as well and the process module 7.1.3. InitModel is obligatory for initialization (cf. section 7. Submodels for details).

There are five classes of process modules: *Initial processes*, *Microbial processes, Resource processes*, *Global processes*, and *Postprocessing modules*. Upon starting a model simulation, the *Initial processes* are executed once to initialize the model (cf. section 7.1. Initial processes). For example, the process module 7.1.2. InitCluster positions all microbes in the center of the environment to simulate a community that starts growing from a single aggregate. All other process modules are executed repeatedly throughout the simulation. However, they are not necessarily always executed at once as different process modules may use different time step lengths (cf. Fig 1). *Microbial processes* are executed for each microbe (cf. section 7.2. Microbial processes). *Resource processes* are executed for each spatial grid cell and each resource, respectively (cf. section 7.3. Resource processes). *Global processes* are used when access to a single microbe or resource grid cell is not sufficient to simulate the process (cf. section 7.4. Global processes). For example, 7.4.1 Diffusion is implemented as a *Global process* instead of as a *Resource process* because the algorithm requires access to the entire array of grid cells. The latter three types of process modules are assumed to run simultaneously and are thus updated accordingly (cf. below). *Postprocessing modules* (cf. section 7.5 Postprocessing modules) are executed at the end of a time step after all other process modules (that were to be executed in that time step). They correct for undesired effects, such as the spatial overlap of microbial individuals (cf. section 7.5.2. Shoving).

Each process module (except for *Initial processes*) is defined with an individual time step dt. Thus, different processes can be modeled with different temporal resolution. They are only executed if their specific time step *dt* is an integer divisor of the current time *t* (Fig 1).

Process modules have read-only access to the current values of all state variables. The change ∂_p that a process needs to apply to a state variable V is stored in a temporary variable $\tilde{V} = \partial_p(V)$. Note that each process module defines its own $\tilde{V}$. After a set of simultaneously running processes is completely executed, all state variables V are updated according to

$$V(\tau + 1) = V(\tau) + \sum \tilde{V},$$

where τ indicates the state of the modeled system before the update and $\tau + 1$ after the update. The sum is taken over all $\tilde{V}$ from different process modules. The decoupling of computation and updating is necessary to ensure synchronous updates of state variables when processes are assumed to run simultaneously.

Throughout the simulation, the time variable is iteratively incremented by the smallest possible time step $1\ mT$. Fig 1 illustrates the procedure following the increment of the time variable in an exemplary model. The simulation stops if either the time variable or the number of microbes exceeds the predefined respective maximum value and optionally if the number of microbes becomes zero.

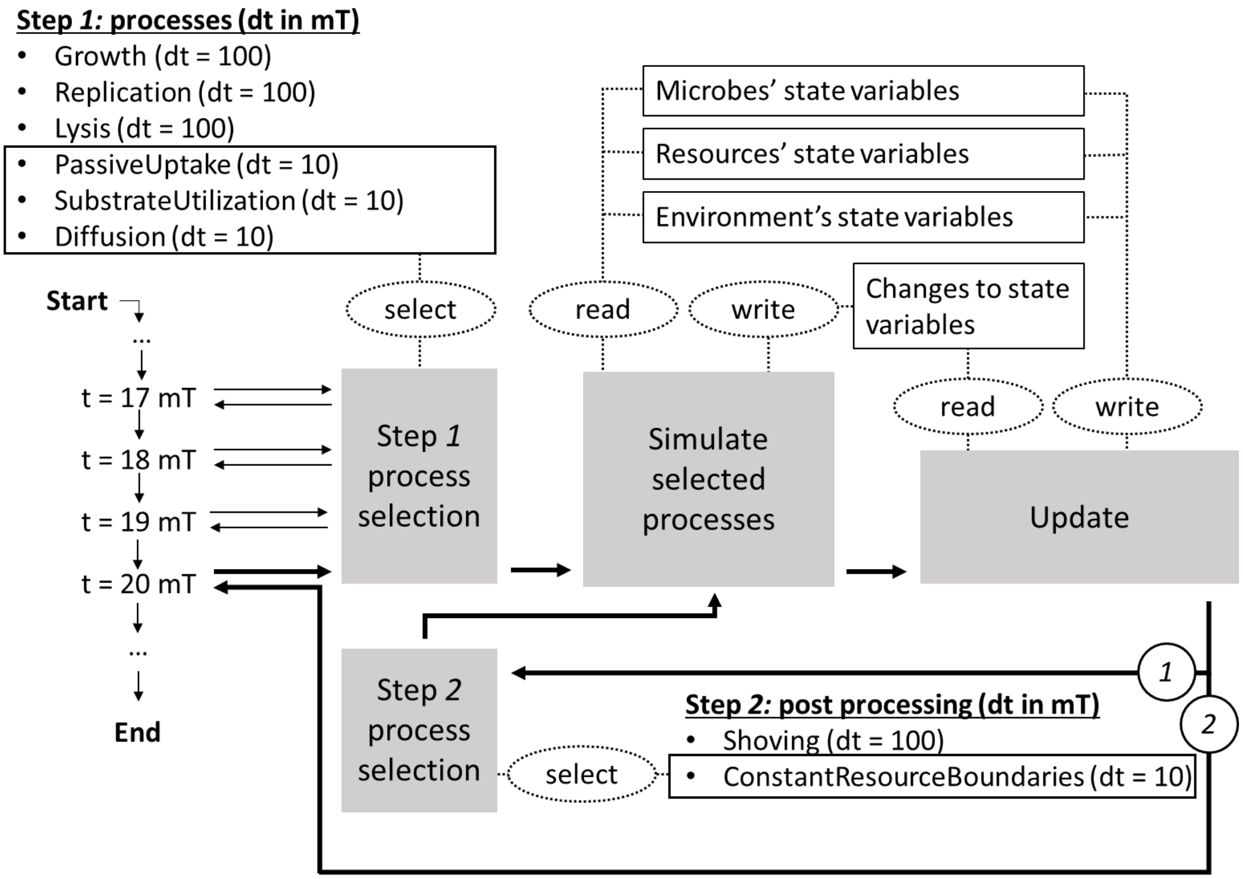

Fig 1. Process scheduling in McComedy. The simulation is organized in an iterative workflow. After incrementing the time variable process modules are checked whether they are ready for execution, i.e. if their specific time step dt is an integer divisor of the current time. If no process modules are ready for execution the iteration is over and the time variable is incremented again. If any process modules are ready for execution they can read the entities' state variables and execute their algorithms. Resulting changes to the state variables are written into temporary variables. After that, a synchronous update is applied by adding the values of the temporary variables to the state variables of the entities. Then (following arrow number 1) it is checked if any Postprocessing modules are ready for execution. If so, the selected Postprocessing modules are executed in the same manner as the process modules before. After updating the changes made by the Postprocessing modules, or if no Postprocessing modules needed to be executed, (following arrow number 2) the iteration is over and the time variable is incremented again.

Due to synchronized access to state variables by several entities and processes, it can happen that a state variable is changed beyond physically meaningful values (e.g. a negative resource concentration). If this happens the model is reset to the previous state and the simulation is continued with tenfold shorter time steps (ceiled to mT) of all process modules. If all process modules are ran with the minimal time step $1\ \mathrm{mT}$ and the problem still occurs, the simulation is stopped with an error message.

4. Design concepts

4.1. Basic principles

IBMs created with McComedy represent the interaction of microbes and resources under consideration of space and physical and biological processes. The underlying idea is that IBMs can be designed by combining distinct pre-implemented processes. This is possible because the pre-implemented processes are independent of assumptions that are specific to certain systems and therefore generic. Different

processes are assumed to act on different temporal scales and can therefore be modeled with different time step lengths. This is possible due to the strict modularization of the processes (cf. section 3. Process overview and scheduling).

4.2. Emergence

Emerging patterns from IBMs created with McComedy depend on the specific design. Usually, the abundance of microbes of different types as well as spatial patterns constitute key results of the simulations. They emerge on the basis of local interactions and metabolic parameters rather than being imposed by superordinate rules. Assumed initial patterns such as microbes being located in a cluster or biofilm can be imposed by the initial positioning.

4.3. Adaptation

The microbes are assumed to make no individual decisions regarding their behavior. For instance, microbes consume as much resources as possible in order to increase biomass. However, in evolutionary terms, adaptation plays a critical role in the model as the best adapted individuals will benefit from their advantages in terms of survival or reproduction and eventually dominate the community.

4.4. Objectives

As there is no direct adaptive behavior, objectives for decision-making are not required.

4.5. Learning

No learning mechanisms are implemented.

4.6. Prediction

As there is no direct adaptive behavior, predictions for decision-making are not required.

4.7. Sensing

If resource uptake is implemented in a model, microbes are assumed to sense local concentrations of the resources which they can consume.

4.8. Interaction

If uptake and release of resources is modeled, direct interaction takes place between microbes and the environment as the microbes consume or release resources. Also, microbes directly compete with each other for these resources and for space. The consumption and release of resources can mediate cooperative interaction between microbes of different types.

4.9. Stochasticity

Stochasticity is used in the model initialization. Microbes are placed randomly in the simulated environment whereby the X, Y, and Z positions are drawn from a uniform distribution. Further stochasticity is used in the process modules 7.2.3. ChangeGenotype, 7.2.5. Flow, 7.1.2. InitCluster, 7.2.8. Lysis and 7.2.11. Replication (cf. section 7. Submodels for implementation details).

4.10. Collectives

For some model designs, simulations will result in the emergence of collectives in the form of one or several spatial aggregates of microbes.

4.11. Observation

For each simulation run the model creates two result files, one for the state of each microbe and one for the concentration profiles of every resource. All state variables that are used in the selected process modules are written into the result files. These are updated in predefined time intervals (which can vary between the two files). The result data is compiled as tables in long format and saved as .txt files.

5. Initialization

Upon simulation start, five parameter files which define the model by listing the (1) process modules, (2) resource parameters, (3) microbe parameters, (4) general parameters, and (5) settings are read in and the environment is instantiated according to the specified spatial extent (cf. section 2. Entities, state variables, and scales). The parameter files are .txt files that contain tables with parameter names and values in separate columns (separated by tab stops). It is strongly recommended to create the parameter files using the graphical user interface of McComedy (Fig 2). Further initialization is performed by the *InitialProcess* modules (cf. section 7.1. Initial processes).

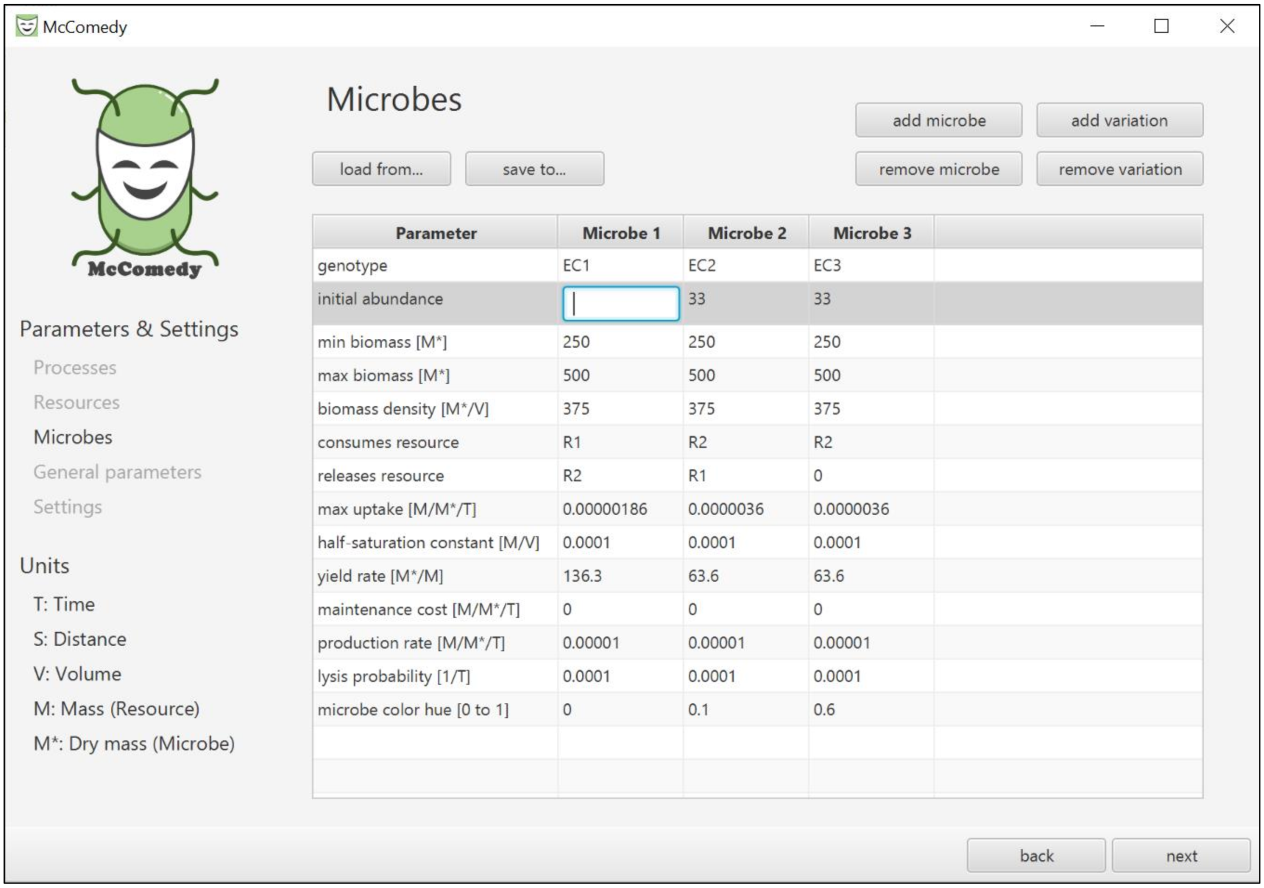

Fig 2. Screenshot of the graphical user interface of McComedy. According to the selected processes, necessary parameters are listed. For each type of microbe, the user can edit the parameter values.

6. Input data

The model does not use input data to represent time-varying processes.

7. Submodels

Since McComedy is a generic modeling platform, the following process modules (i.e. the submodels) to be combined in one specific simulation model can be flexibly selected. They are grouped into five classes: *Initial processes*, *Microbial processes*, *Resource processes*, *Global processes*, and *Postprocessing modules*. As process modules do not modify state variables directly, changes are written into temporary variables which are indicated with '~' (cf. Section 3. Process overview and scheduling for details on the updating scheme for synchronous processes).

7.1. Initial processes

7.1.1. InitBiofilm

If the microbes need to be simulated as a biofilm, this process module can be used. The Y-position of all microbes will be set to a value defined by the global parameter *biofilm Y-position*, resulting in an initial spatial distribution on a plane orthogonal to the Y-axis. When simulating biofilms it is recommended to use this process module together with 7.2.7 ImpermeableMicrobeBoundaries with the parameter values *impermeable Y-boundaries* true and *impermeable boundary offset* equal to *biofilm Y-position*.

Table 2. Parameters for process module InitBiofilm

**Parameter**	**Dimension**	**Type**	**Variable type**	**Explanation**
biofilm Y-position	S	global parameter	Decimal	Position of the bottom of the biofilm on the Y-axis

7.1.2. InitCluster

If the microbes need to be initialized in an aggregated state, as opposed to free floating planktonic microbes, this process module can be used. All microbes will be positioned close to the center of the simulated environment, within the radius

$$r_{init} = r_{m0}\sqrt[3]{N},$$

where r_{m0} is the radius of the first microbe in the list of all microbes and N is the initial number of microbes.

7.1.3. InitModel

This process module is necessary in every IBM in McComedy. Therefore, the user is not supposed to add it to the model manually. Instead, it is integrated automatically. According to the values of the necessary parameters (Table 3), the microbes and resources are added to the environment. The positions of the microbes are drawn randomly from a uniform distribution in the range of the entire environment.

Table 3. Parameters for process module InitModel

**Parameter**	**Dimension**	**Type**	**Variable type**	**Explanation**
spatial extent X	S	global parameter	Integer	Size of environment in X-direction

spatial extent Y	S	global parameter	Integer	Size of environment in Y-direction
spatial extent Z	S	global parameter	Integer	Size of environment in Z-direction
simulation time	T	global parameter	Decimal	Maximum time before simulation stops
stop when all microbes die	-	global parameter	Boolean	Indicates whether simulation should be stopped when no microbes are contained in the environment
max microbes number	-	global parameter	Integer	If the number of microbes exceeds this value the simulation is stopped
constant initial position	-	global parameter	Boolean	Defines whether the initial microbe positions should be the same across replicates and variations
random generator seed	-	global parameter	Integer	Seed for random generator. If it is 0 a random seed is generated
genotype	-	microbe parameter	String	The name of a microbial type (e.g. strain or species). It serves as an identifier
min biomass	M*	microbe parameter	Decimal	The minimum dry mass of a microbe
max biomass	M*	microbe parameter	Decimal	The maximum dry mass of one microbe
initial abundance	-	microbe parameter	Integer	Initial number of microbes of respective type
biomass density	M^*/S^3	microbe parameter	Decimal	Biomass density of microbe as dry mass per volume
microbe color hue	-	microbe parameter	Decimal	Between 0 and 1. Color hue of respective type in visual model output as defined by HSB color representation
resource name	-	resource parameter	String	The name of this resource. It serves as an identifier
initial concentration	M/ S^3	resource parameter	Decimal	The initial concentration in each grid cell
resource color hue	-	resource parameter	Decimal	Between 0 and 1. Color hue of resource in visual model output as defined by HSB color representation
max render concentration	-	resource parameter	Decimal	Maximum concentration to which resource concentrations are scaled in visual model output. Concentrations equal to or above this maximum are rendered at full brightness

7.2. Microbial processes

7.2.1. Attachment

This process module allows microbes to attach to each other. Which types of microbes are compatible to attach to each other is defined by the microbe parameter *attach to genotype*. Each microbe checks if other microbes of a compatible type are located within the *attachment distance*. If so, these other microbes get attached to the focal microbe and are added to its list *Attached microbes*. The position of every microbe in the environment is changed according to

$$\tilde{X} = d_x\, c_a\, dt,$$

$$\tilde{Y} = d_y\, c_a\, dt,$$

$$\tilde{Z} = d_z\, c_a\, dt,$$

where $\tilde{X}$, $\tilde{Y}$, and $\tilde{Z}$ are the temporary variables for the changes made by this process module with respect to the spatial axes X, Y, and Z. d_x, d_y, and d_z are the X, Y, and Z components of the mean directional vector from the focal microbe to all attached microbes and c_a is the microbe parameter *attachment coefficient*, which describes how tightly microbes are attached to each other (i.e. how fast they are pulled together if separated by a distance shorter than the *detachment distance*).

If attached microbes are separated further away than the *detachment distance* they become detached from the focal microbe and are removed from its list *Attached microbes*.

Table 4. Parameters for process module Attachment

**Parameter**	**Dimension**	**Type**	**Variable type**	**Explanation**
attach to genotype	-	microbe parameter	String	The type of microbes that can attach to this type. Multiple types can be defined, separated by ';'. The string 'all' indicates that all types attach to this type
attachment distance	S	microbe parameter	Decimal	Defines how close Microbes need to be in order to attach. The distance is measured between the microbes' surfaces
detachment distance	S	microbe parameter	Decimal	Defines how far attached Microbes need to be forced away from each other in order to detach
attachment coefficient	1/T	microbe parameter	Decimal	Between 0 and 1. Defines how much a microbe is pulled towards its attached neighbors per time unit T, whereby 1 means the microbe is pulled completely towards the attached microbes and 0 means it is not pulled at all

Table 5. State variables added by process module Attachment

State variable	Type	Variable type	Explanation
Attached microbes	Microbe state variable	List of Microbes	Lists all microbes that are currently attached to the focal microbe

Table 6. Dependencies of process module Attachment

Dependency	Explanation
ProximityManager	The computationally intensive test which microbes are close enough for attachment requires information on which microbes are in each other's vicinity, provided by *ProximityManager*

7.2.2. CellPartition

This process module estimates in a computationally efficient way how much a microbe (with its spherical shape) overlaps with the spatial grid cells of the environment. To this end, the grid cell that overlaps with the microbe's midpoint and all 26 neighboring grid cells are considered. First, for each of these 27 grid cells, a grid cell is considered to overlap with the microbe if the microbes' radius is larger than the shortest distance between its midpoint and the grid cell. Second, for all grid cells that overlap with the microbe, the reciprocal values of the squared distances between the grid cell's midpoint and the microbe's midpoint are computed and these values are normalized such that their sum equals 1. Thus, each value estimates the relative overlap of the microbe with the respective grid cell.

Table 7. State variables added by process module CellPartition

State variable	Type	Variable type	Explanation
Overlaps	Microbe state variable	3D-Array of size 3x3x3	Contains the estimate how much the microbe overlaps with each of the adjacent resource grid cells

7.2.3. ChangeGenotype

This process module allows microbes changing their genotype. When the genotype of the microbe is changed, all parameter values that are associated with the new genotype (as defined in the microbes parameter file) are changed, accordingly. The probability of changing the genotype is defined by the microbe parameter *change genotype probability*. The genotype that the microbe is changed to is defined by the microbe parameter *change genotype to*.

Table 8. Parameters for process module ChangeGenotype

Parameter	Dimension	Type	Variable type	Explanation
change genotype to	-	microbe parameter	String	The type of microbe this microbe is changed to. The string must equal a microbe's genotype parameter
change genotype probability	1/T	microbe parameter	Decimal	The probability at which a microbe's type is changed

7.2.4. ConstantProduction

This process module simulates production of resource R in the intracellular product pool P_R according to

$$\tilde{P}_R = B\, p_R\, dt,$$

where $\tilde{P}_R$ is the temporary variable for the changes made by this process module with respect to the intracellular product pool P_R, B is the biomass of the microbe and p_R is the microbe's *production rate* of resource R. The production is constant and thus independent of other processes (e.g. the consumption of resources).

Table 9. Parameters for process module ConstantProduction

Parameter	Dimension	Type	Variable type	Explanation
production rate	M/M*/T	microbe parameter	Decimal	The rate at which resources are produced

Table 10. State variables added by process module ConstantProduction

State variable	Type	Variable type	Explanation
Product pool	Microbe state variable	Decimal	Intracellular pool of resources that have been produced by this microbe

7.2.5. Flow

This process module simulates microbial flow in any direction. The flow of the microbes is computed by

$$\tilde{X} = (v_x + \varepsilon_x)\, dt,$$

$$\tilde{Y} = \left(v_y + \varepsilon_y\right) dt,$$

$$\tilde{Z} = (v_z + \varepsilon_z)\, dt,$$

where $\tilde{X}$, $\tilde{Y}$, and $\tilde{Z}$ are the temporary variables for the changes made by this process module, v_x, v_y, and v_z are the components of the velocity vector as defined by the global parameters *mean flow X*, *mean flow Y*, and *mean flow Z*, and ε_x, ε_y, and ε_z are the components of a random vector drawn from a normal distribution with a mean of zero and standard deviations corresponding to the global parameters *flow SD X*, *flow SD Y*, and *flow SD Z*. Note that the resources are not affected by this module.

Table 11. Parameters for process module Flow

Parameter	Dimension	Type	Variable type	Explanation
mean flow X	S/T	global parameter	Decimal	The velocity at which microbes move in X direction
mean flow Y	S/T	global parameter	Decimal	The velocity at which microbes move in Y direction
mean flow Z	S/T	global parameter	Decimal	The velocity at which microbes move in Z direction
flow SD X	S/T	global parameter	Decimal	Standard deviation of movement in X direction
flow SD Y	S/T	global parameter	Decimal	Standard deviation of movement in Y direction
flow SD Z	S/T	global parameter	Decimal	Standard deviation of movement in Z direction

7.2.6. Growth

Biomass growth of the microbes is modeled by

$$\tilde{B} = y\,G,$$

$$\tilde{G} = -G,$$

where $\tilde{B}$ and $\tilde{G}$ are the temporary variables for the changes made by this process module with respect to the biomass B and the amount G of resources that has been allocated to growth. y is the microbe parameter *yield*.

Table 12. Parameters for process module Growth

Parameter	Dimension	Type	Variable type	Explanation
yield	M*/M	microbe parameter	Decimal	The rate at which resources are transformed into biomass

Table 13. State variables added by process module Growth

**State variable**	**Type**	**Variable type**	**Explanation**
Growth resources	Microbe state variable	Decimal	Resources allocated to biomass growth

7.2.7. ImpermeableMicrobeBoundaries

For processes that affect the position of the microbes, this process module allows to change from default periodic boundary conditions to closed boundary conditions, separately for each spatial dimension. This can be controlled by the parameters *impermeable X-boundaries*, *impermeable Y-boundaries*, and *impermeable Z-boundaries*, respectively. This is implemented as follows: Microbes that approach the impermeable boundaries closer than the value of the parameter *impermeable boundary offset* are moved away from the boundary such that their distance to the boundary equals the value of the parameter *impermeable boundary offset*. It is therefore important to set a large enough offset (the modeler needs to estimate how much a microbe can move within one time step) because if a microbe moves far enough to cross the actual border of the environment within a time step, its new location will be computed according to the default periodic boundaries.

Table 14. Parameters for process module Flow

**Parameter**	**Dimension**	**Type**	**Variable type**	**Explanation**
impermeable X-boundaries	-	global parameter	Boolean	Indicates whether X-boundaries are impermeable
impermeable Y-boundaries	-	global parameter	Boolean	Indicates whether Y-boundaries are impermeable
impermeable Z-boundaries	-	global parameter	Boolean	Indicates whether Z-boundaries are impermeable
impermeable boundary offset	S	global parameter	Decimal	The distance from the impermeable point to the true boundary

7.2.8. Lysis

This process module simulates cell death by lysis. For each microbe type the probability of lysis is defined by the parameter *lysis probability*. Individuals die randomly according to this probability and are removed from the environment.

Table 15. Parameters for process module Lysis

Parameter	Dimension	Type	Variable type	Explanation
lysis probability	1/T	microbe parameter	Decimal	Between 0 and 1. Indicates the probability of a cell lysing

7.2.9. PassiveRelease

This process module simulates release of intracellular products into the environment. As microbes have spherical shapes with explicit sizes and continuous spatial positions, it can happen that an individual overlaps with several discrete grid cells of the environment. Therefore, at each time step, the resources contained in the *Product pool* P_R are distributed among the grid cells that the microbes overlaps with, according to

$$\tilde{C}_{R,x,y,z} = B\, a_{x,y,z}\, P_R,$$

where $\tilde{C}_{R,x,y,z}$ is the temporary variable for the changes made by this process module with respect to $C_{R,x,y,z}$, the amount of resource R in the grid cell at position X, Y, Z. B is the biomass of the microbe and $a_{x,y,z}$ is the proportional overlap of the microbe with the grid cell at position X, Y, Z. The entire *Product pool* is consequently depleted:

$$\tilde{P}_R = -P_R.$$

Here $\tilde{P}_R$ is the temporary variable for changes made by this process module with respect to the *Product pool* P_R. Approximate proportions of the overlaps of the microbes with different grid cells are computed by the process module 7.2.2 CellPartition.

Table 16. Parameters for process module PassiveRelease

Parameter	Dimension	Type	Variable type	Explanation
releases resource	-	microbe parameter	String	The resource that is released by respective type. The string must equal a resource's name parameter

Table 17. Dependencies of process module PassiveRelease

Dependency	Explanation
ConstantProduction	Produced resources are stored in the state variable *Product pool* which is added by the process module *ConstantProduction*
CellPartition	Resources are released into overlapping grid cells proportionally to the overlap. The overlap is estimated by *CellPartition*

7.2.10. PassiveUptake

This process module simulates the consumption of resources from the environment by microbes. As microbes have continuous spatial coordinates while resources are discretized to a grid, it can happen that a microbe overlaps with several grid cells. Resources from grid cells that the microbe overlaps with are transferred into the intracellular pool S_R of resource R according to Monod-kinetics

$$\tilde{S}_R = B\, q\, dt = B \sum a_{x,y,z} \frac{q_{max} C_{R,x,y,z}}{K_M + C_{R,x,y,z}}\, dt,$$

$$\tilde{C}_{R,x,y,z} = -B\, a_{x,y,z}\, q\, dt,$$

where $\tilde{S}_R$ and $\tilde{C}_{R,x,y,z}$ are temporary variables for the changes made by this process module with respect to the intracellular pool S_R and $C_{R,x,y,z}$ is the amount of resource R in the grid cell at position X, Y, Z. B is the biomass of the microbe, a_{xyz} is the proportional overlap of the microbe with the grid cell at position X, Y, Z, q_{max} is the maximum uptake rate, and K_M is the Monod half-saturation constant.

Approximate proportions of the overlaps of the microbes with different grid cells are computed by the process module 7.2.2 CellPartition.

Table 18. Parameters for process module PassiveUptake

**Parameter**	**Dimension**	**Type**	**Variable type**	**Explanation**
consumes resource	-	microbe parameter	String	The resource that is consumed by respective type. The string must equal a resource's name parameter
half-saturation constant	M/S³	microbe parameter	Decimal	Resource concentration in the vicinity of the microbe at which the uptake rate is half maximal (according to the microbe parameter *max uptake*)
max uptake	M/M*/T	microbe parameter	Decimal	Theoretical maximum uptake rate of resource

Table 19. Dependencies of process module PassiveUptake

**Dependency**	**Explanation**
SubstrateUtilization	Consumed resources are stored in the state variable *Substrate pool* which is added by the process module *SubstrateUtilization*
CellPartition	Resources are consumed from overlapping grid cells proportionally to the overlap. The overlap is estimated by *CellPartition*

7.2.11. Replication

Replication via cell division is modeled by this process module. Upon reaching a critical biomass, as defined by the microbe parameter *maximum biomass*, the microbe's biomass is divided by two and another microbe with identical state variable values is added to the environment. As the two microbes occupy the

exact same location, the newly added microbe is shifted in a random direction by a very small distance (i.e. < 0.0001 S). This allows the process module 7.5.2. Shoving to operate and minimize the overlap of both microbes.

7.2.12. Starvation

This process module simulates death due to starvation. If a microbe's state variable *Starvation* indicates starving, the individual is removed from the environment.

Table 20. Dependencies of process module Starvation

**Dependency**	**Explanation**
SubstrateUtilization	The state variable *Starvation* that indicates whether a microbe is starving is added by the process module *SubstrateUtilization*

7.2.13. SubstrateUtilization

This process module allocates consumed resources S_R to maintenance and growth. The amount of substrate allocated to maintenance M is given by

$$M = -B\, m\, dt,$$

where B is the biomass of the microbe and m is the microbe parameter *maintenance cost*. If this amount exceeds S_R, this microbe is flagged as *starving* (see also 7.2.12. Starvation). Otherwise, the remainder of S_R is transferred to the microbe's state variable *Growth resources* (G), that is to the pool of resources allocated to biomass growth

$$\tilde{G} = \max(S_R - M, 0),$$

$$\tilde{S}_R = -S_R\, .$$

Here $\tilde{G}$ and $\tilde{S}_R$ are the temporary variables for the changes made by the process modules with respect to the *Growth resources* G and the consumed resources S_R. Note that the chosen implementation of maintenance results in a restriction regarding the choice of time step lengths. The time step of the process module 7.2.10. PassiveUptake, which provides resources into the *Substrate pool,* is supposed to be smaller than or equal to the time step of SubstrateUtilization. If it were chosen larger and, thus, SubstrateUtilization executed multiple times between executions of 7.2.10. PassiveUptake, the entire *Substrate pool* would be depleted during the first execution and hence the maintenance would be impossible to satisfy in subsequent executions. However, this restriction does not apply if the *maintenance cost* is set to zero.

Table 21. Parameters for process module SubstrateUtilization

**Parameter**	**Dimension**	**Type**	**Variable type**	**Explanation**
maintenance cost	M/M*/T	microbe parameter	Decimal	The amount diverted into maintenance from the *Substrate pool*

Table 22. State variables added by process module SubstrateUtilization

State variable	Type	Variable type	Explanation
Substrate pool	Microbe state variable	Decimal	Intracellular pool of resources that have been consumed but not utilized yet
Starvation	Microbe state variable	Boolean	Indicates whether the microbe is starving

Table 23. Dependencies of process module SubstrateUtilization

Dependency	Explanation
Growth	Resources allocated to growth are stored in the state variable *Growth resources* which is added by the process module *Growth*

7.3. Resource processes

7.3.1. ResourceDecay

This process module simulates decay of resources over time in the environment. The concentration of the resources is reduced in every position by the respective *decay rate*.

Table 24. Parameters for process module ResourceDecay

Parameter	Dimension	Type	Variable type	Explanation
decay rate	1/T	resource parameter	Decimal	Between 0 and 1. Defines the rate at which each resource decays in the environment

7.4. Global processes

7.4.1. Diffusion

Diffusion of resources is modeled according to Fick's second law of diffusion. We applied the finite difference method in three dimensions as described by Muglar and Scott [3] for two dimensions, which results in

$$\tilde{C}_{R,x,y,z} = D_R \, \frac{dt}{dS^2} \left(C_{R,x+dS,y,z} + C_{R,x-dS,y,z} + C_{R,x,y+dS,z} + C_{R,x,y-dS,z} + C_{R,x,y,z+dS} + C_{R,x,y,z-dS} - 6\, C_{R,x,y,z} \right),$$

where $\tilde{C}_{R,x,y,z}$ is the temporary variable for changes made by this process module with respect to $C_{R,x,y,z}$, the concentration of resource R in position X, Y, Z, D_R is the diffusion constant of that resource according to the parameter *diffusion constant*, and dS is the distance between the midpoints of two adjacent

resource grid cells which is always 1 S. The solution of the equation above is unstable if $D_R * dt > \frac{1}{6}$, hence the parameter *diffusion constant* and the time step dt must be sufficiently small.

Table 25. Parameters for process module Diffusion

Parameter	Dimension	Type	Variable type	Explanation
diffusion constant	S^2/T	resource parameter	Decimal	Diffusion constant

7.4.2. LocalSource

This process module allows to introduce local sources or sinks of resources. The positions of these points are defined by the resource parameters *local source X*, *local source Y*, and *local source Z*. The parameter *local source type* specifies whether the resource concentration as defined by the resource parameter *local source concentration* is set only at simulation start ('once'), in each time step ('set'), or the defined concentration is added in each time step ('add'). When the *local source type* is set to 'add', a negative value can be chosen in order to simulate a sink. However, this can result in a negative local resource concentration which will cause the simulation to stop with a warning message. Therefore, it is better to simulate a sink by using 'set' to 0 (or also a low positive value), if this is reasonable in the modeled context.

Table 26. Parameters for process module LocalSource

Parameter	Dimension	Type	Variable type	Explanation
has local source	-	resource parameter	Boolean	Indicates whether this resource has any local sources
local source X	S	resource parameter	Decimal	X position(s) of local source(s). If multiple sources exist, the positions are separated by whitespaces ' '
local source Y	S	resource parameter	Decimal	Y position(s) of local source(s). If multiple sources exist the positions are separated by whitespaces ' '
local source Z	S	resource parameter	Decimal	Z position(s) of local source(s). If multiple sources exist, the positions are separated by whitespaces ' '
local source concentration	M/S^3	resource parameter	Decimal	Concentration(s) of local source(s). If multiple sources exist, the concentrations are separated by whitespaces ' '
local source type	-	resource parameter	String	'add', 'set', or 'once'. Indicates, whether the concentration of each source is added to the respective grid cell each

				time step (add), held constant (set), or set once upon model initialization

7.4.3. ProximityManager

This process module discretizes the space of the simulated environment into raster cells of a size defined by the global parameter *proximity raster cell size* and groups all microbes together that overlap to the greatest extent with the same raster cell. The estimate of which microbes are in rough vicinity to a given microbe consists of all microbes in the same raster cell and all 26 adjacent raster cells (Note that the proximity raster cells may differ from the resource grid cells, which are always of size 1 S^3). This increases computational efficiency for process modules that simulate local interaction between microbes (e.g. 7.2.1. Attachment, 7.5.2. Shoving).

Table 27. Parameters for process module ProximityManager

**Parameter**	**Dimension**	**Type**	**Variable type**	**Explanation**
proximity raster cell size	S	global parameter	Integer	The length of raster cells that are used to group microbes into discrete spatial positions

7.5. Postprocessing modules

7.5.1. ConstantResourceBoundaries

This process module allows to change from the default periodic boundary conditions for processes that affect resource concentrations to constant boundary conditions, separately for each spatial dimension. This can be controlled with the parameters *constant X-boundaries*, *constant Y-boundaries*, and *constant Z-boundaries*, respectively. On execution of this process module, if applied to the corresponding dimension, all resource amounts in the outermost grid cells are set to the value of *concentration at boundary*.

Table 28. Parameters for process module ConstantResourceBoundaries

**Parameter**	**Dimension**	**Type**	**Variable type**	**Explanation**
constant X-boundaries	-	resource parameter	Boolean	Indicates whether X-boundaries are constant
constant Y-boundaries	-	resource parameter	Boolean	Indicates whether Y-boundaries are constant
constant Z-boundaries	-	resource parameter	Boolean	Indicates whether Z-boundaries are constant
concentration at boundary	M/S^3	resource parameter	Decimal or String	If Decimal, the concentration that is maintained at fixed boundaries. If String "as initial", the resource parameter value *initial concentration* is used, which

				is convenient when the initial concentration is varied

7.5.2. Shoving

Shoving describes the process of the microbes pushing each other away if they overlap, also referred to as spatial relaxation. The shoving algorithm from iDynoMiCS (Algorithm S1 in [4]) is used. It is not always possible to get rid of any overlap between microbes in reasonable computational time (the algorithm stops after 12 iterations), therefore the process aims to minimize the total overlap as much as possible.

Table 29. Dependencies of process module Shoving

**Dependency**	**Explanation**
ProximityManager	The test which microbes overlap can be computationally boosted if information which microbes are close to each other is provided. This is done by *ProximityManager*

References

1. Grimm V, Berger U, Bastiansen F, Eliassen S, Ginot V, Giske J, et al. A standard protocol for describing individual-based and agent-based models. Ecological Modelling. 2006;198(1-2): 115-26. doi: 10.1016/j.ecolmodel.2006.04.023.
2. Grimm V, Railsback SF, Vincenot CE, Berger U, Gallagher C, DeAngelis DL, et al. The ODD protocol for describing agent-based and other simulation models: a second update to improve clarity, replication, and structural realism. Journal of Artificial Societies and Social Simulation. 2020;23(2). doi: 10.18564/jasss.4259.
3. Mugler DH, Scott RA. Fast fourier transform method for partial differential equations, case study: the 2-D diffusion equation. Computers & Mathematics with Applications. 1988;16(3): 221-8. doi: 10.1016/0898-1221(88)90182-4.
4. Lardon LA, Merkey BV, Martins S, Dotsch A, Picioreanu C, Kreft JU, et al. iDynoMiCS: next-generation individual-based modelling of biofilms. Environmental Microbiology. 2011;13(9): 2416-34. doi: 10.1111/j.1462-2920.2011.02414.x.
